# Web-Based Cancer Symptom Self-Management System: A Randomized Clinical Trial

**DOI:** 10.1001/jamanetworkopen.2025.8353

**Published:** 2025-05-05

**Authors:** David Cella, Nicola Lancki, Maja Kuharic, Betina Yanez, Michael Bass, Martha G. Garcia, Kimberly A. Webster, Justin D. Smith, Mary O’Connor, Ava Coughlin, September Cahue, Sheetal Kircher, Ann Marie Flores, Frank J. Penedo, Roxanne E. Jensen, Ashley Wilder Smith, Kimberly Richardson, Cynthia Barnard, Christopher M. George, Dean G. Tsarwhas, Denise Scholtens, Sofia F. Garcia

**Affiliations:** 1 Department of Medical Social Sciences, Northwestern University Feinberg School of Medicine, Chicago, Illinois; 2 Department of Preventive Medicine, Division of Biostatistics, Northwestern University Feinberg School of Medicine, Chicago, Illinois; 3 Department of Population Health Science, Division of Health System Innovation and Research, Spencer Fox Eccles School of Medicine at the University of Utah, Salt Lake City; 4 Northwestern Medicine Division of Hematology and Oncology, Department of Medicine, Northwestern University Feinberg School of Medicine, Chicago, Illinois; 5 Cancer Survivorship Institute, Robert H. Lurie Comprehensive Cancer Center of Northwestern University, Chicago, Illinois; 6 Department of Psychology and Medicine, University of Miami, Miami, Florida; 7 Outcomes Research Branch, Health Care Delivery Research Program, National Cancer Institute, Rockville, Maryland; 8 Black Cancer Collaborative, Chicago, Illinois

## Abstract

**Importance:**

Patients with cancer and cancer survivors frequently experience symptoms that increase the need for health care services and impair quality of life. Effective symptom management is critical for comprehensive patient-centered cancer care.

**Objective:**

To evaluate the effectiveness of adding a bilingual (English and Spanish), web-based self-management program to an electronic health record (EHR)–integrated patient-reported outcome for cancer (cPRO) assessment in reducing symptom burden and health care resource use (HCRU).

**Design, Setting, and Participants:**

This patient-level randomized clinical trial was performed at the Northwestern Memorial HealthCare system in Chicago, Illinois. Participants included 1614 adult patients with cancer or cancer survivors in 30 clinics who were enrolled between April 1, 2020, and April 8, 2023, and followed up for 12 months until May 8, 2024.

**Interventions:**

Usual care (UC) consisting of an EHR-integrated cPRO assessment or enhanced care (EC), which offered an additional tailored web-based self-management program.

**Main Outcomes and Measures:**

Patient-Reported Outcomes Measurement Information System measures of anxiety, depression, fatigue, pain interference, and physical function collected at baseline and monthly for 12 months. Secondary outcomes included HCRU measures (inpatient and/or observation visits and days, emergency department and/or urgent care visits, and days of hospital stay).

**Results:**

A total of 1614 patients were included in the analysis, with 804 randomized to EC and 810 to UC. The mean (SD) age was 61 (13) years; 1095 patients (67.8%) were female. Only 419 EC participants (52.1%) accessed the website, with only 197 (47%) returning; the median time per visit was 45 seconds (IQR, 45-105 seconds). There were no statistically significant differences between EC and UC across the cPRO outcomes over 12 months. The mean change from baseline at each assessment time point for treatment effects (EC vs UC) ranged from −0.19 (95% CI, −0.86 to 0.33; *P* = .64) for physical function to 0.11 (95% CI, −0.75 to 0.79; *P* = .87) for fatigue. Zero-inflated negative binomial and logistic regression models showed no significant differences in HCRU outcomes: inpatient and/or observation visits (incidence rate ratio [IRR], 0.90; 95% CI, 0.72-1.12), emergency department and/or urgent care visits (IRR, 0.99; 95% CI, 0.84-1.16), and days of hospital stay (IRR, 1.05; 95% CI, 0.83-1.33).

**Conclusions and Relevance:**

In this randomized clinical trial, adding a bilingual web-based self-management program to EHR-integrated cPRO did not reduce symptom burden or HCRU compared with cPRO alone. Low engagement with the web-based program highlights the need for strategies to enhance engagement and tailor interventions to those who would benefit most.

**Trial Registration:**

ClinicalTrials.gov Identifier: NCT03988543

## Key Points

**Question:**

Can a web-based enhanced care (EC) self-management program improve patient-reported outcomes (PROs) and reduce health care resource use (HCRU) compared with usual care (UC)?

**Findings:**

In this randomized clinical trial of 1614 patients with cancer and cancer survivors, no significant differences were found between EC and UC groups in symptom burden or HCRU during 12 months of follow-up. Only 419 of 804 participants (52%) in the EC group ever visited the website, with only 197 (47%) returning, indicating limited engagement.

**Meaning:**

These findings suggest that this specific intervention approach should not be implemented without redesign focusing on clinical workflow integration and other strategies to increase patient engagement.

## Teaser

This randomized clinical trial assesses the effectiveness of enhanced care, including a bilingual web-based self-management program, compared with usual care in reducing symptom burden for patients with cancer and cancer survivors.

## Introduction

Patients with cancer and cancer survivors experience a range of symptoms and adverse effects that can significantly impact their quality of life and overall well-being.^1^ Despite advances in cancer treatment, effective symptom management remains a critical component of comprehensive cancer care.^2,3^ Inadequate symptom control can lead to decreased treatment adherence, increased health care resource use (HCRU), reduced survival, and diminished quality of life.^4,5^ Therefore, there is a pressing need for innovative, patient-centered interventions that enable timely and effective symptom monitoring and management.

Patient-reported outcomes (PROs) have become a valuable tool in cancer care, providing crucial insights into patients’ symptoms, functional status, and quality of life.^6^ Using PROs in clinical practice can improve communication between patients and their clinicians, help detect unrecognized symptoms, guide treatment decisions, and ultimately improve patient outcomes.^7,8^ Moreover, collecting and integrating PROs into the electronic health record (EHR) can allow for real-time symptom monitoring and management, enabling timely intervention when needed.^9^ Basch et al^5^ found that routine PRO symptom monitoring during cancer treatment improved overall survival compared with usual care (UC). Similarly, Denis et al^10^ found that a web-based PRO system for symptom monitoring in patients with lung cancer led to improved survival and quality of life. This approach can improve the quality and efficiency of cancer care delivery and reduce health care costs by preventing unnecessary emergency department visits and hospitalizations.^4,11^

The rapid growth of digital health technologies has opened new ways to provide supportive care interventions to patients with cancer without significantly increasing clinician burden.^12^ EHR-integrated PROs have shown promise in improving symptom management and patient outcomes.^13,14^ Furthermore, patient-centered websites and smartphone applications that provide tailored symptom management information and resources have been known to improve patient engagement, self-efficacy, and outcomes.^15,16^ These digital platforms can offer personalized symptom tracking, education, and self-care strategies, helping patients to take a more active role in managing their symptoms.^17^ These interventions show promise, and their effectiveness may hinge on patient engagement. Previous studies have shown variable adoption rates of such tools, ranging from 10% to 60% among patients with chronic conditions, including cancer.^18^ Understanding engagement patterns is important for developing and implementing effective digital health interventions in cancer care.^19^

The Northwestern University Improving the Management of Symptoms During and Following Cancer Treatment (NU IMPACT) study examined the effectiveness of offering a patient-centered website in English and Spanish to support symptom self-management in patients with cancer.^20^ This study builds on previous work that implemented EHR-integrated PROs for cancer (cPRO) symptom monitoring program across a multisite health care system,^20^ enabling systematic assessment of patient-reported symptoms and supportive care needs using validated measures such as the Patient-Reported Outcomes Measurement Information System (PROMIS).^21^

The primary objective of this study was to assess the effectiveness of enhanced care (EC) in reducing symptom burden for patients with cancer and cancer survivors compared with UC. UC included cPRO symptom monitoring and clinician alerts, while EC also offered a bilingual, web-based, tailored self-management program (MyNM Care Corner; Northwestern Memorial HealthCare). This trial evaluated whether adding web-based self-management tools provided benefits beyond standard cPRO symptom monitoring alone, rather than testing basic cPRO effectiveness. The secondary objective was to evaluate the impact of the EC intervention on HCRU compared with UC. We hypothesized that EC would more effectively reduce symptom burden and optimize HCRU than UC alone.

## Methods

This randomized clinical trial was approved by the Northwestern University Institutional Review Board, and all participants provided written informed consent via REDCap surveys. This study followed the Consolidated Standards of Reporting Trials Extension (CONSORT Extension) reporting guideline.

### Study Design and Participants

NU IMPACT included a patient-level randomized clinical trial, conducted in the postimplementation period of a larger stepped-wedge cluster-randomized trial, to evaluate the effectiveness of an EC intervention (with access to self-management resources) compared with UC (which included clinician alerts for severe symptoms and endorsed needs) in reducing symptom burden among patients with cancer and cancer survivors.^22,23^ In the overall study design, 7 clusters (consisting of 30 outpatient adult oncology clinics within the Northwestern Medicine health care system) were assigned to launch preimplementation phase data collection at quarterly intervals, and then each cluster crossed over to the postimplementation phase after 6 months.^24^ Participants were enrolled during both study phases (staggered by cluster) to complete regular cPRO assessments. Consented patients in the postimplementation period were randomized 1:1 to EC or UC. Detailed protocols of the larger stepped-wedge trial and patient-randomized clinical trial have been published previously^20^; this report focuses on the patient-level randomized clinical trial evaluation of effectiveness outcomes for EC vs UC. Adult patients and survivors (aged ≥18 years) with a diagnosis of a solid tumor or hematologic malignant neoplasm within the past 10 years were eligible if they met the following criteria: (1) treatment or survivorship care at participating clinics, (2) a clinic visit within the past year at one of the recruiting sites, (3) their clinician agreed for them to be contacted about the study, (4) ability to read English or Spanish, and (5) a valid email address.^21,25^ We included patients across the cancer care continuum because symptom burden and self-management needs persist beyond active treatment. In addition, we opted to test the potential benefit of a low-cost, web-based symptom management application across our health system. Exclusion criteria were severe cognitive impairment or current participation in another symptom management trial. Patients who consented to participate in the study were randomized to EC vs UC. Randomization was stratified by cluster, gender or sex, the EHR oncology treatment intent at the time of eligibility, and study language (English or Spanish). Race and ethnicity data were extracted from the electronic health record. Race categories included American Indian or Alaska Native, Asian or Native Hawaiian or Other Pacific Islander, Black or African American, White, and unknown or not reported; ethnicity categories included Hispanic or Latino, non-Hispanic or non-Latino, and unknown or not reported. These data were collected because of an interest in promoting health equity for all patients, regardless of race or ethnicity. An EHR medical oncology module (Beacon; Epic Systems Corporation) was used to determine whether patients had a curative (curative, adjuvant, or neoadjuvant) or noncurative (palliative or maintenance) care plan intent at enrollment, assigning them to the respective care continuum group. Patients without an EHR treatment plan were placed in the group with survivorship or no treatment plan.

### Interventions

The EC intervention included a web-based self-management program (MyNM Care Corner) in addition to EHR-integrated cPRO monitoring, while UC consisted of routine cPRO monitoring (with clinical alerts) alone.^4,10^ The cPRO system uses PROMIS measures to assess key symptoms (pain interference, fatigue, anxiety, depression, and physical function) and supportive care needs.^25,26,27^ Assessments are completed via the EHR patient portal or in the clinic. Results were scored in real time, and those meeting or exceeding severity thresholds triggered an email alert for clinician intervention.^5^ The self-management program provides symptom education and management strategies and resources in English and Spanish and is tailored to each patient’s specific needs.^16,28^ For example, if a patient endorsed moderate-to-severe anxiety on their cPRO assessment, the self-care program website would post information on anxiety management strategies to their personalized dashboard. Patients accessed the platform via a customized link, optimized for desktop and mobile web browsers, provided through study emails. The website content aimed to improve patients’ knowledge about cancer-related concerns, develop self-management skills for symptoms, and empower motivation for self-care. Implementation strategies focused on patient engagement through personalized outreach, including emails, phone calls, and quarterly illustrated newsletters that highlighted symptom management content and encouraged website use. Designated clinicians were notified via EHR in-basket messaging when any supportive care need was endorsed or the reported symptom exceeded the preset threshold for severity, even if the others did not exceed the threshold. For fatigue, options to refer to light exercise or physical therapy were provided. Anxiety threshold scores, like depression threshold scores, were directly triaged to mental health clinicians.

### Outcomes and Assessments

The primary effectiveness outcomes were PROMIS measures for anxiety, depression, fatigue, pain interference, and physical function, assessed at baseline and monthly for up to 12 months after enrollment in the study.^21,29,30^ One advantage of PROMIS measures is the use of computer adaptive testing, where items are selected from a large data bank and tailored to each individual based on their initial responses, and tailored short forms that are scored on the same metric. PROMIS measure scores are standardized to the US general population with a mean of 50 and SD of 10, with higher scores indicating greater symptom severity or burden.^31,32^

Secondary outcomes included HCRU (emergency department visits, inpatient and/or observation visits, and inpatient length of stay), measured at sites within the same health system collected over a 12-month period. These outcomes were captured through the EHR for visits within Northwestern Medicine.

Data on EC engagement were collected over a 12-month study period, focusing on 3 metrics: the number of participants accessing the website (defined as those who logged into the website at least once), the frequency of visits (the number of times each participant accessed the website), and the duration of visits (the time spent on the website per visit).

### Statistical Analysis 

The primary analysis estimated the mean difference in longitudinal PROMIS symptom scores (anxiety, depression, fatigue, pain interference, and physical function) collected monthly from baseline to 12 months for the EC vs UC groups. Patients were included in the primary analysis if they completed at least 1 cPRO during the study. Linear mixed models were used for analysis, specifying fixed effects for treatment assignment, randomization strata variables (cluster, treatment intent, gender, and language preference), baseline cPRO value, and quarter of enrollment. Individual-level random effects for the intercepts and slopes and an autoregressive covariance structure were included to account for the dependence of within-individual cPRO measurements over time.^33^ The step-down Dunnett method was used to adjust for multiple comparisons across the 5 primary outcomes.^34,35^ Estimated treatment effects, 95% CIs, and adjusted *P* values were reported. Mean differences in change from baseline to 6 and 12 months for EC vs UC were also evaluated using linear regression models with treatment arm (EC vs UC) as the primary covariate of interest and the same model covariates used for the longitudinal mixed models.

Zero-inflated negative binomial regression models were used to analyze the secondary count outcomes (number of inpatient and/or observation visits and days and emergency department and/or urgent care visits) during 12 months, as these outcomes exhibited excess zeros and overdispersion.^36^ Negative binomial models were used to analyze days of hospital stay. For a subset of participants in clusters 1 and 2 (central and Chicago regions), logistic regression models were used to analyze the occurrence of at least 1 visit to the oncology triage clinic unique to that region. This analysis was conducted separately due to the limited availability of this service. All models included the same covariates for the primary outcomes. While the trial was designed and powered to detect differences in the primary cPRO outcomes, it was not specifically powered to detect differences in HCRU.

Missing data were handled using multiple imputations by joint modeling under the missing-at-random assumption using R package jomo, version 4.1.0 (R Program for Statistical Computing).^37,38^ Model estimates were pooled for statistical inference using Rubin rules, and statistical significance was assessed at a 2-sided α level of .05. All analyses were conducted using R, version 4.1.0, with the nlme package for linear mixed models,^39^ the glmmTMB package for zero-inflated negative binomial models,^40^ and the jomo package for multiple imputations.^40,41,42^ The full trial protocol and statistical analysis plan are available in Supplement 1.

## Results

### Participant Characteristics

**Figure 1.**
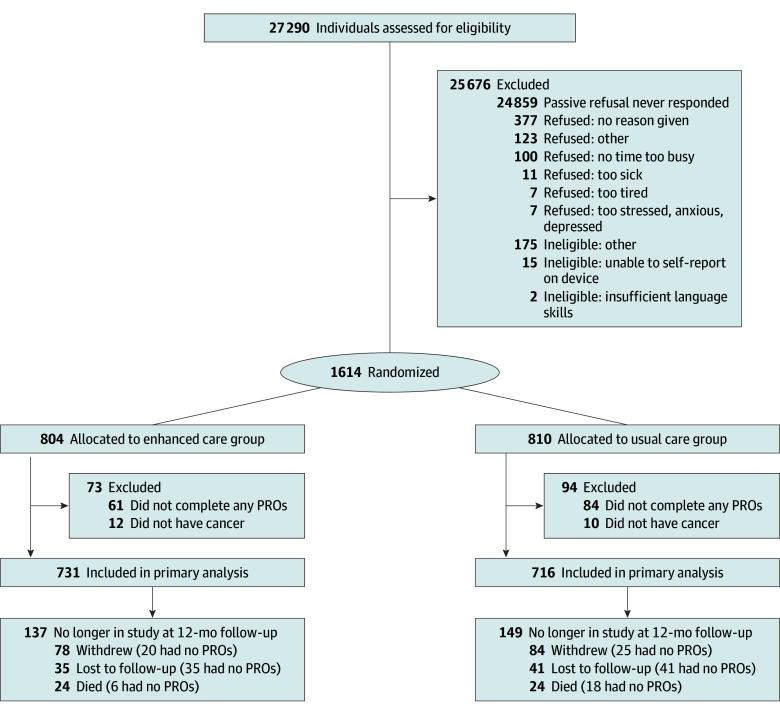
Participants in the Northwestern University Improving the Management of Symptoms During and Following Cancer Treatment Study Flowchart PRO indicates patient-reported outcome.

Of 1614 participants enrolled between April 1, 2020, and April 8, 2023 (mean [SD] age, 61 [13] years; 1095 [67.8%] female and 519 [32.2%] male), 804 were randomized to EC and 810 to UC (Figure 1 and eTable 4 in Supplement 2). A total of 78 patients (4.8%) were Hispanic or Latino. Two patients (0.1%) were American Indian or Alaska Native, 55 (3.4%) were Asian or Native Hawaiian or Other Pacific Islander, 101 (6.3%) were Black or African American, 1315 (81.5%) were White, and 141 (8.7%) had unknown or unreported race. One thousand thirty participants (70.0%) were in survivorship with no active treatment plan. The most common cancer types were breast (485 [30.5%]), colorectal and other gastrointestinal tract (284 [17.8%]), and lymphoma (189 [11.9%]). Baseline characteristics were well-balanced between the 2 study arms (Table 1). Among those randomized, 1447 were included in primary analyses (22 were found not to have cancer after randomization, and 145 did not complete at least 1 cPRO over the study period and were considered lost to follow-up). Participants of the noncompletion group were younger (median age, 57 [IQR, 42-68] vs 62 [IQR, 52-71] years), more likely to have Spanish as their language preference (4 of 145 [2.8%] vs 17 of 1447 [1.2%]), and less likely to have experience completing cPRO prior to their enrollment in the study (56 of 145 [38.6%] vs 915 of 1447 [63.2%]). Of the 145 participants lost to follow-up, 25 (17.2%) were found to be either deceased or withdrew due to reasons such as being too sick, tired, or busy. Follow-up was completed May 8, 2024.

**Table 1.** Participant Characteristics^a^

Characteristic	Participant group			
	Usual care (n = 810)	Enhanced care (n = 804)	All (N = 1614)
Age, mean (SD), y^b^	61 (13)	60 (13)	61 (13)
Sex^b^			
Female	549 (68)	546 (67.9)	1095 (67.8)
Male	261 (32)	258 (32.1)	519 (32.2)
Gender identity^c^			
Women	472 (67.7)	488 (66.7)	960 (67.2)
Men	223 (32.0)	242 (33.1)	465 (32.5)
Transgender man	0	0	0
Transgender woman	0	1 (0.1)	1 (0.1)
Other	2 (0.3)	1 (0.1)	3 (0.2)
Race^b^			
American Indian or Alaska Native	2 (0.2)	0	2 (0.1)
Asian or Native Hawaiian or Other Pacific Islander	29 (3.6)	26 (3.2)	55 (3.4)
Black or African American	43 (5.3)	58 (7.2)	101 (6.3)
White	659 (81.4)	656 (81.6)	1315 (81.5)
Unknown or not reported	77 (9.5)	64 (8.0)	141 (8.7)
Ethnicity^b^			
Hispanic or Latino	41 (5.1)	37 (4.6)	78 (4.8)
Non-Hispanic or non-Latino	592 (73.1)	602 (74.9)	1194 (74.0)
Unknown or not reported	177 (21.9)	165 (20.5)	342 (21.2)
Study language^b^			
English	797 (98.4)	791 (98.4)	1588 (98.4)
Spanish	13 (1.6)	13 (1.6)	26 (1.6)
Employment status^d^			
Full-time employed	230 (32.9)	275 (37)	505 (35.2)
Part-time employed	64 (9.2)	64 (8.7)	128 (8.9)
Retired	272 (38.9)	257 (35)	529 (37.2)
On disability or on leave of absence	66 (9.4)	75 (10)	141 (9.8)
Homemaker	31 (4.4)	31 (4.2)	62 (4.3)
Unemployed or full-time student only	28 (4.0)	28 (3.8)	56 (3.9)
Prefer not to answer	8 (1.1)	5 (0.7)	13 (0.9)
Educational level^e^			
High school or less	65 (9.4)	55 (7.5)	120 (8.4)
Some college, technical, or associate degree	183 (26.4)	173 (23.7)	356 (25.0)
College graduate	224 (32.3)	245 (33.6)	469 (33.0)
Graduate or advanced degree	221 (31.9)	257 (35.2)	478 (33.6)
Insurance type^f^			
Medicare only	45 (6.5)	45 (6.1)	90 (6.3)
Medicaid	28 (4.0)	27 (3.7)	55 (3.8)
Private	370 (53.1)	403 (55.0)	773 (54.1)
Uninsured or self-pay	4 (0.6)	7 (1.0)	11 (0.8)
I do not know	9 (1.3)	14 (1.9)	23 (1.6)
Medicare Advantage	236 (33.9)	233 (31.8)	469 (32.8)
Military or veterans sponsored	5 (0.7)	4 (0.5)	9 (0.6)
Treatment intent			
Curative	157 (19.4)	158 (19.7)	315 (19.5)
Noncurative	86 (10.6)	83 (10.3)	169 (10.5)
No treatment plan	567 (70.0)	563 (70.0)	1130 (70.0)
Time since diagnosis, mean (SD), y^g^	3.3 (4.5)	3.1 (4.4)	3.2 (4.5)
Cancer type^h^			
Breast	238 (29.8)	247 (31.2)	485 (30.5)
Central nervous system or brain	5 (0.6)	2 (0.3)	7 (0.4)
Colon or rectal	63 (7.9)	51 (6.4)	114 (7.2)
Other gastrointestinal tract	78 (9.8)	92 (11.6)	170 (10.7)
Head or neck	5 (0.6)	13 (1.6)	18 (1.1)
Lung or thoracic	28 (3.5)	24 (3.0)	52 (3.3)
Sarcoma or bone	8 (1.0)	12 (1.5)	20 (1.3)
Melanoma	24 (3.0)	22 (2.8)	46 (2.9)
Nonmelanoma skin	2 (0.3)	1 (0.1)	3 (0.2)
Ovary	22 (2.8)	22 (2.8)	44 (2.8)
Cervix	3 (0.4)	4 (0.5)	7 (0.4)
Endometrial (uterus)	32 (4.0)	29 (3.7)	61 (3.8)
Other gynecologic	4 (0.5)	4 (0.5)	8 (0.5)
Prostate	26 (3.3)	20 (2.5)	46 (2.9)
Other genitourinary	24 (3.0)	22 (2.8)	46 (2.9)
Leukemia	69 (8.6)	65 (8.2)	134 (8.4)
Lymphoma	89 (11.1)	100 (12.6)	189 (11.9)
Other hematologic	60 (7.5)	51 (6.4)	111 (7.0)
Other	1 (0.1)	0	1 (0.1)
Multiple malignant neoplasms	12 (1.5)	7 (0.9)	19 (1.2)
Endocrine	7 (0.9)	4 (0.5)	11 (0.7)
Cancer stage^i^			
0	22 (4.2)	21 (4.0)	43 (4.1)
I	210 (39.6)	210 (40.4)	420 (40.0)
II	109 (20.6)	102 (19.6)	211 (20.1)
III	102 (19.2)	95 (18.3)	197 (18.8)
IV	87 (16.4)	92 (17.7)	179 (17.0)
Metastatic^j^	166 (20.5)	160 (19.9)	326 (20.0)
Charlson Comorbidity Index, mean (SD)	5.9 (3.2)	5.7 (3.0)	5.8 (3.1)

^a^ Data are presented as No. (%) of participants unless noted otherwise.

^b^ Extracted from the Northwestern Medicine Enterprise Data Warehouse (EDW).

^c^ There were 185 missing responses or missing in EDW.

^d^ There were 180 missing responses or missing in EDW.

^e^ There were 191 missing responses or missing in EDW.

^f^ There were 184 missing responses or missing in EDW.

^g^ There were 395 missing responses or missing in EDW.

^h^ There were 22 missing responses or missing in EDW.

^i^ There were 564 missing responses or missing in EDW.

^j^ Defined as stage IV or palliative treatment intent.

Of the 804 participants randomized to the EC group, only 419 (52.1%) accessed the website at least once during the 12-month study period and only 197 (47.0%) returned. Among those who accessed the site, the median number of visits was 1 (IQR, 1-2), with a median duration of 45 seconds (IQR, 45-105 seconds) per visit. Fatigue, physical function, and pain consistently ranked among the most frequently accessed topics within the self-management program symptom library. Engagement challenges reported included technical barriers such as needing to access the site through personalized email links rather than direct website access, and the absence of clinical workflow integration. A detailed analysis of factors affecting engagement will be reported separately. Mean number of assessments completed over the study period by group is provided in eTable 3 in Supplement 2, with proportions illustrated in eFigure in Supplement 2. Engagement with the underlying cPRO system was similar between groups, with 505 of 804 participants in the EC group (62.8%) and 509 of 810 participants in the UC arms (62.8%) completing at least 1 assessment during follow-up (median, 1; IQR, 0-3). Clinical severity alert rates from the cPRO system were also comparable between groups, with 186 EC participants (23.1%) and 177 UC participants (21.9%) generating at least 1 severe symptom alert during follow-up (*P* > .90).

### Primary Outcome

**Figure 2.**
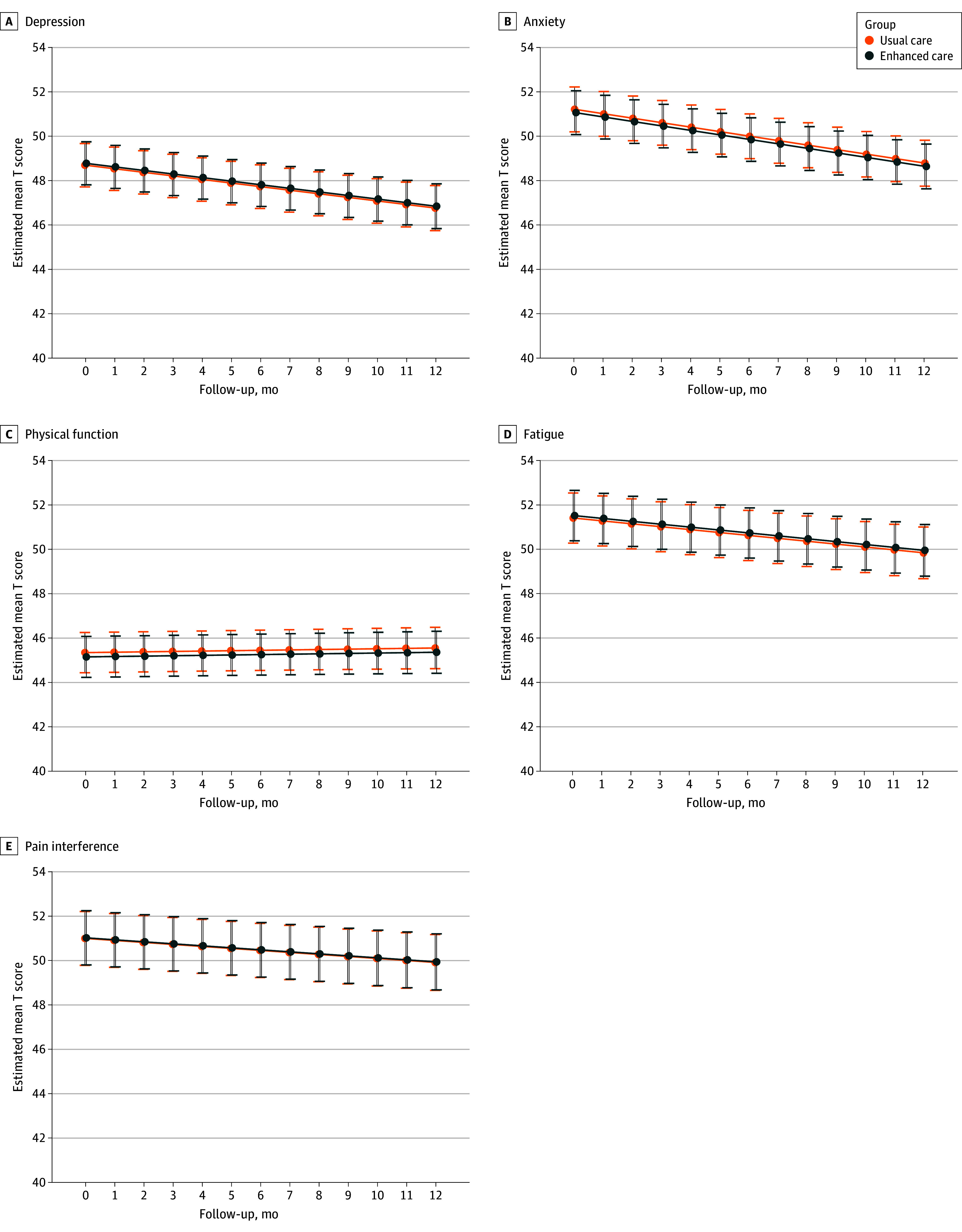
Patient-Reported Outcomes Measurement Information System (PROMIS) Score Distribution and Model-Based Mean Change From Baseline at Each Assessment Time Point Calculated as least square mean estimates of T score over time by treatment group, based on a linear mixed model adjusting for site (cluster), cancer type, gender, language preference, baseline value of PROMIS (domain), and quarter of enrollment. Smooth trajectories reflect modeled means rather than observed means. Bars represent 95% CIs.

Primary outcome results from the linear-mixed model analysis are provided in eTable 2 in Supplement 2. Linear mixed models, adjusted for stratification variables and baseline cPROs, indicated no statistically significant differences between the EC and UC groups across the 5 PROMIS domains over 12 months. Estimated mean treatment effects (EC vs UC) were depression (0.08; 95% CI, −0.62 to 0.64; *P* = .87), anxiety (−0.15; 95% CI, −0.90 to 0.44; *P* = .79), physical function (−0.19; 95% CI, −0.86 to 0.33; *P* = .64), fatigue (0.11; 95% CI, −0.75 to 0.79; *P* = .87), and pain interference (0.03; 95% CI, −0.86 to 0.72; *P* = .91) **(**Figure 2 and Table 2).

**Table 2.** Primary Outcome Results From Linear Mixed Models: Difference in Mean Change From Baseline at Each Assessment Time Point, Baseline to 12 Months^a^

PRO domain	Treatment effect, estimate (95% CI)	*P* value
Depression T score	0.08 (−0.62 to 0.64)	.87
Anxiety T score	−0.15 (−0.90 to 0.44)	.79
Physical function T score	−0.19 (−0.86 to 0.33)	.64
Fatigue T score	0.11 (−0.75 to 0.79)	.87
Pain interference T score	0.03 (−0.86 to 0.72)	.91

Abbreviation: PRO, patient-reported outcome.

^a^ Data were adjusted for multiple comparisons using Dunnett step-down procedure and were determined using a model adjusted for sex, therapeutic intent category, cluster, study language, baseline PRO value, and quarter enrolled.

eTable 1 in Supplement 2 presents the mean changes in PROMIS scores from baseline to 6 and 12 months for the EC and UC groups. At 6 months, there were no statistically significant differences between the 2 groups for any of the PROMIS outcomes, including estimates for depression (−0.11; 95% CI, −0.85 to 0.62; *P* = .76), anxiety (0.17; 95% CI, −0.61 to 0.96; *P* = .67), physical function (0.31; 95% CI, −0.34 to 0.97; *P* = .35), fatigue (−0.26; 95% CI, −1.17 to 0.64; *P* = .57), and pain interference (−0.40; 95% CI, −1.34 to 0.55; *P* = .41). Similarly, at 12 months, no significant differences remained between the EC and UC groups on any PROMIS domain. Analysis stratified by cancer continuum (curative, noncurative, or survivorship) showed no significant differences in outcomes between groups in any stratum (eTable 6 in Supplement 2).

### Secondary Outcomes

The EC intervention did not significantly reduce HCRU compared with UC during 12 months of follow-up (Table 3 and eTable 5 in Supplement 2). Models showed no significant differences between the EC and UC groups in the global summary of HCRU (incidence rate ratio [IRR], 0.96; 95% CI, 0.84-1.12; *P* = .63), number of hospital admissions (IRR, 0.90; 95% CI, 0.72-1.12; *P* = .35), number of emergency department or urgent care visits (IRR, 0.99; 95% CI, 0.84-1.16; *P* = .88), days of hospital stay (IRR, 1.05; 95% CI, 0.83-1.33; *P* = .65), or in any oncology triage clinic (odds ratio, 2.21; 95% CI, 0.44-16.22; *P* = .36).

**Table 3.** Health Care Resource Use Outcomes Over 12 Months by Study Arm^a^

Outcome	6-mo EC vs UC		12-mo EC vs UC	
	Treatment effect (95% CI)	*P* value	Treatment effect (95% CI)	*P* value
Global summary, IRR^b^	1.05 (0.89-1.26)	.55	0.96 (0.84-1.12)	.63
Hospital admissions, IRR	0.94 (0.72-1.22)	.64	0.90 (0.72-1.12)	.35
ED or urgent care visits, IRR	1.10 (0.92-1.33)	.30	0.99 (0.84-1.16)	.88
Any OTC visits, OR^c^	2.21 (0.44-16.22)	.36	2.21 (0.44-16.22)	.36
Hospital days of stay, IRR	1.02 (0.77-1.34)	.91	1.05 (0.83-1.33)	.65

Abbreviations: EC, enhanced care; ED, emergency department; IRR, incidence rate ratio; OR, odds ratio; OTC, oncology triage clinic; UC, usual care.

^a^ Data were determined using a model adjusted for sex, therapeutic intent category, cluster, study language, and quarter enrolled.

^b^ The sum of hospital admissions and ED or urgent care visits.

^c^ The 6-month and 12-month estimates are identical because all OTC visits occurred within the first 6 months of the study period.

## Discussion

This study evaluated the effectiveness of offering a web-based self-management program (MyNM Care Corner) in addition to an EHR-integrated cPRO symptom monitoring system (including clinical alerts) in reducing symptom burden and HCRU among patients with cancer and cancer survivors compared with cPRO symptom monitoring alone. Despite the known potential benefits of web-based self-management support in enhancing patient engagement and self-care,^43^ we found no differences in primary outcomes between those patients randomly assigned to care enhanced with a web-based self-management program compared with usual oncology care. The trial was designed and powered to detect differences in the primary cPRO outcomes; it was not specifically powered to detect differences in HCRU. The high proportion of patients without active treatment plans (70%) raises the question of whether focusing on patients during early active treatment phases, when symptom management needs are typically higher, might yield different results. However, our stratified analyses showed no significant intervention effects, even in the subgroup receiving active treatment.

We believe that 2 key covariates contributed to the lack of demonstrated clinically important benefits in this setting and can inform future interventions and research. First, the cPRO symptom monitoring system, which was available to both the EC and UC groups, may have lessened the potential benefit of the additive support since it already provided a foundation for symptom management by facilitating timely clinical responses to symptoms of concern to patients (primarily for those with severe symptoms that triggered clinical alerts). The self-management resource was designed to provide support for patients who were symptomatic but did not reach the threshold for severe symptoms and clinical alerts. However, the addition of this self-management resource may not have offered sufficient incremental benefit over the cPRO system alone to be discernible in results. Previous studies^5,10^ have shown that EHR-integrated symptom monitoring can improve patient outcomes and survival. For example, in 2006, Basch et al^5^ conducted a randomized clinical trial showing that cPRO symptom monitoring improved health-related quality of life and survival in patients with advanced cancer. Similarly, Denis et al^10^ found that web-based follow-up improved survival compared with routine surveillance in patients with lung cancer.

Second, and likely as important, the lack of significant differences between the EC and UC groups may be largely explained by low engagement with the self-management resource. With only 52% of EC participants accessing the website and the time spent there being brief, the potential impact of the intervention was almost certainly not fully realized. The low engagement with the self-management resource underscores the difficulty of promoting patient engagement and self-management in digital health interventions, which often limits their impact on health outcomes.^44,45^ Several factors may have contributed to the low uptake and use of the self-management resource, such as lack of technological access, limited digital literacy, competing priorities in life, or patients’ perceived lack of relevance or usefulness.^46,47^ Access to the website presented challenges: the link was customized for each individual and provided only via email. Feedback suggested that requiring personalized password-protected links for security was a key barrier to access and engagement. Although efforts were made to keep patients engaged through emails, phone calls, and newsletters that reshared personalized links, participants had to refer to these materials to access the site if they had not bookmarked it. The resource’s lack of integration within existing patient portals or applications likely reduced its visibility and ease of access. Moreover, patient activation and self-efficacy play a role in adopting and sustaining self-management interventions.^11,48,49^ Self-management behaviors and activation levels among patients with cancer can vary widely depending on their individual characteristics, disease stage, and treatment phase.^50,51^ The limited engagement likely reflects barriers in our implementation approach. Future interventions should consider integrating self-management tools into routine clinic workflows and providing initial face-to-face orientation to the website, rather than relying solely on patient initiative and remote access. Additionally, focusing on patients with higher symptom management needs, particularly those in active treatment phases, may improve uptake and utility of such tools. The current approach of providing website access without active clinical integration may have been insufficient to promote sustained engagement.

Future research should explore specific barriers and facilitators to patient engagement with web-based self-management interventions in cancer care. This includes developing and evaluating strategies for more seamless and frictionless access and personalized content. Our experience and patient reports indicate that patients are more likely to use a resource when it is directly recommended to them by their principal oncology clinician, which is challenging in the context of already crowded appointments. Future studies should investigate the optimal integration of these interventions with existing clinical workflows and patient care pathways without overburdening clinicians. This may involve identifying ideal subpopulations who may benefit most, fully integrating with existing portals, improving technical strategies such as applications and text messaging, determining optimal timing and frequency of self-management support, and finding effective ways to coordinate self-management interventions with ongoing clinical care and symptom monitoring.

Last, the COVID-19 pandemic may have further compounded the challenges of patient engagement and self-management in cancer care.^52,53^ For instance, the shift toward telemedicine and virtual care during the pandemic may have altered patient engagement and health care–seeking behaviors, potentially masking the intervention’s effects.^54^ Additionally, psychological distress, social isolation, and competing demands experienced by many patients with cancer during the pandemic could have impacted their motivation and capacity for self-management.^55^

### Limitations

Several limitations of this study should be noted. First, the study was conducted within a single health care system, which may limit the applicability of the findings to other settings with different patient populations, resources, or clinical workflows. Second, there is potential for selection bias because the consented population may systematically differ from those who did not participate in the study. Third, HCRU data are restricted to use of the Northwestern Medicine health care system only. The large patient sample, recruited from multiple oncology clinics, is a strength of the study that enhances the generalizability of the findings across economic, urban and rural, race and ethnicity, and language characteristics. However, this health system has a modest level of about 5% Spanish-speaking and -reading participants.

## Conclusions

In this randomized clinical trial, the availability of a web-based self-management program added to an EHR-integrated cPRO did not significantly reduce symptom burden or HCRU among patients with cancer and cancer survivors compared with cPRO alone. However, the low engagement with the web-based program highlights the challenges in implementing digital health interventions. These findings underscore the importance of not only developing effective tools but also ensuring their adoption by patients. Future research should prioritize understanding barriers to engagement and tailoring interventions to those who would benefit most.

## Notes

Supplement 1.
Trial Protocol and Statistical Analysis Plan

Supplement 2. **eTable 1.** Mean Changes in PROMIS Scores From Baseline to 6 and 12 Months **eTable 2.** Primary Outcome Results from Linear-Mixed Model: Including Preimplementation **eTable 3.** Mean Numbers Completed Over Study Period by Group **eTable 4.** Primary Outcome Numbers Completed at Each Time Point by Group **eTable 5.** Health Care Utilization Outcomes and Treatment Effects at 6 and 12 Months **eTable 6.** Primary Outcome Results From Linear-Mixed Models Stratified by Beacon Intent **eFigure.** Proportion Completed at Each Time Point by Group

Supplement 3.
Data Sharing Statement

## References

[1] Reilly CM, Bruner DW, Mitchell SA, . A literature synthesis of symptom prevalence and severity in persons receiving active cancer treatment. Support Care Cancer. 2013;21(6):1525-1550. doi:10.1007/s00520-012-1688-0 23314601 PMC4299699

[2] Burkett VS, Cleeland CS. Symptom burden in cancer survivorship. J Cancer Surviv. 2007;1(2):167-175. doi:10.1007/s11764-007-0017-y 18648958

[3] Cleeland CS. Symptom burden: multiple symptoms and their impact as patient-reported outcomes. J Natl Cancer Inst Monogr. 2007;(37):16-21. doi:10.1093/jncimonographs/lgm005 17951226

[4] Basch E, Deal AM, Kris MG, . Symptom monitoring with patient-reported outcomes during routine cancer treatment: a randomized controlled trial. J Clin Oncol. 2016;34(6):557-565. doi:10.1200/JCO.2015.63.0830 26644527 PMC4872028

[5] Basch E, Deal AM, Dueck AC, . Overall survival results of a trial assessing patient-reported outcomes for symptom monitoring during routine cancer treatment. JAMA. 2017;318(2):197-198. doi:10.1001/jama.2017.7156 28586821 PMC5817466

[6] Kyte D, Duffy H, Fletcher B, . Systematic evaluation of the patient-reported outcome (PRO) content of clinical trial protocols. PLoS One. 2014;9(10):e110229. doi:10.1371/journal.pone.0110229 25333349 PMC4198237

[7] Kotronoulas G, Kearney N, Maguire R, . What is the value of the routine use of patient-reported outcome measures toward improvement of patient outcomes, processes of care, and health service outcomes in cancer care? a systematic review of controlled trials. J Clin Oncol. 2014;32(14):1480-1501. doi:10.1200/JCO.2013.53.5948 24711559

[8] Chen J, Ou L, Hollis SJ. A systematic review of the impact of routine collection of patient reported outcome measures on patients, providers and health organisations in an oncologic setting. BMC Health Serv Res. 2013;13:211. doi:10.1186/1472-6963-13-211 23758898 PMC3700832

[9] Jensen RE, Snyder CF, Abernethy AP, . Review of electronic patient-reported outcomes systems used in cancer clinical care. J Oncol Pract. 2014;10(4):e215-e222. doi:10.1200/JOP.2013.001067 24301843 PMC4094646

[10] Denis F, Basch E, Septans AL, . Two-year survival comparing web-based symptom monitoring vs routine surveillance following treatment for lung cancer. JAMA. 2019;321(3):306-307. doi:10.1001/jama.2018.18085 30667494 PMC6439676

[11] Lizée T, Basch E, Trémolières P, . Cost-effectiveness of web-based patient-reported outcome surveillance in patients with lung cancer. J Thorac Oncol. 2019;14(6):1012-1020. doi:10.1016/j.jtho.2019.02.005 30776447

[12] Shaffer KM, Turner KL, Siwik C, . Digital health and telehealth in cancer care: a scoping review of reviews. Lancet Digit Health. 2023;5(5):e316-e327. doi:10.1016/S2589-7500(23)00049-3 37100545 PMC10124999

[13] Absolom K, Warrington L, Hudson E, . Phase III randomized controlled trial of eRAPID: eHealth intervention during chemotherapy. J Clin Oncol. 2021;39(7):734-747. doi:10.1200/JCO.20.02015 33417506

[14] Basch E, Barbera L, Kerrigan CL, Velikova G. Implementation of patient-reported outcomes in routine medical care. Am Soc Clin Oncol Educ Book. 2018;38:122-134. doi:10.1200/EDBK_200383 30231381

[15] Børøsund E, Cvancarova M, Ekstedt M, Moore SM, Ruland CM. How user characteristics affect use patterns in web-based illness management support for patients with breast and prostate cancer. J Med Internet Res. 2013;15(3):e34. doi:10.2196/jmir.2285 23454601 PMC3636230

[16] Ruland CM, Maffei RM, Børøsund E, Krahn A, Andersen T, Grimsbø GH. Evaluation of different features of an eHealth application for personalized illness management support: cancer patients’ use and appraisal of usefulness. Int J Med Inform. 2013;82(7):593-603. doi:10.1016/j.ijmedinf.2013.02.007 23507561

[17] Kuijpers W, Groen WG, Aaronson NK, van Harten WH. A systematic review of web-based interventions for patient empowerment and physical activity in chronic diseases: relevance for cancer survivors. J Med Internet Res. 2013;15(2):e37. doi:10.2196/jmir.2281 23425685 PMC3636300

[18] Irizarry T, DeVito Dabbs A, Curran CR. Patient portals and patient engagement: a state of the science review. J Med Internet Res. 2015;17(6):e148. doi:10.2196/jmir.4255 26104044 PMC4526960

[19] Yardley L, Spring BJ, Riper H, . Understanding and promoting effective engagement with digital behavior change interventions. Am J Prev Med. 2016;51(5):833-842. doi:10.1016/j.amepre.2016.06.015 27745683

[20] Cella D, Garcia SF, Cahue S, . Implementation and evaluation of an expanded electronic health record-integrated bilingual electronic symptom management program across a multi-site Comprehensive Cancer Center: the NU IMPACT protocol. Contemp Clin Trials. 2023;128:107171. doi:10.1016/j.cct.2023.107171 36990275 PMC10164083

[21] Cella D, Riley W, Stone A, ; PROMIS Cooperative Group. The Patient-Reported Outcomes Measurement Information System (PROMIS) developed and tested its first wave of adult self-reported health outcome item banks: 2005-2008. J Clin Epidemiol. 2010;63(11):1179-1194. doi:10.1016/j.jclinepi.2010.04.011 20685078 PMC2965562

[22] Curran GM, Bauer M, Mittman B, Pyne JM, Stetler C. Effectiveness-implementation hybrid designs: combining elements of clinical effectiveness and implementation research to enhance public health impact. Med Care. 2012;50(3):217-226. doi:10.1097/MLR.0b013e3182408812 22310560 PMC3731143

[23] Hemming K, Haines TP, Chilton PJ, Girling AJ, Lilford RJ. The stepped wedge cluster randomised trial: rationale, design, analysis, and reporting. BMJ. 2015;350:h391. doi:10.1136/bmj.h391 25662947

[24] Hussey MA, Hughes JP. Design and analysis of stepped wedge cluster randomized trials. Contemp Clin Trials. 2007;28(2):182-191. doi:10.1016/j.cct.2006.05.007 16829207

[25] Garcia SF, Wortman K, Cella D, . Implementing electronic health record-integrated screening of patient-reported symptoms and supportive care needs in a comprehensive cancer center. Cancer. 2019;125(22):4059-4068. doi:10.1002/cncr.32172 31373682 PMC11345862

[26] Wagner LI, Schink J, Bass M, . Bringing PROMIS to practice: brief and precise symptom screening in ambulatory cancer care. Cancer. 2015;121(6):927-934. doi:10.1002/cncr.29104 25376427 PMC4352124

[27] Cella D, Choi SW, Condon DM, . PROMIS^®^ adult health profiles: efficient short-form measures of seven health domains. Value Health. 2019;22(5):537-544. doi:10.1016/j.jval.2019.02.004 31104731 PMC7201383

[28] Berry DL, Hong F, Halpenny B, . Electronic self-report assessment for cancer and self-care support: results of a multicenter randomized trial. J Clin Oncol. 2014;32(3):199-205. doi:10.1200/JCO.2013.48.6662 24344222 PMC3887477

[29] Cleeland CS, Mendoza TR, Wang XS, . Assessing symptom distress in cancer patients: the M.D. Anderson Symptom Inventory. Cancer. 2000;89(7):1634-1646. doi:10.1002/1097-0142(20001001)89:7<1634::AID-CNCR29>3.0.CO;2-V 11013380

[30] Cella D, Gershon R, Lai JS, Choi S. The future of outcomes measurement: item banking, tailored short-forms, and computerized adaptive assessment. Qual Life Res. 2007;16(suppl 1):133-141. doi:10.1007/s11136-007-9204-6 17401637

[31] Cella D, Yount S, Rothrock N, ; PROMIS Cooperative Group. The Patient-Reported Outcomes Measurement Information System (PROMIS): progress of an NIH Roadmap cooperative group during its first two years. Med Care. 2007;45(5)(suppl 1):S3-S11. doi:10.1097/01.mlr.0000258615.42478.55 17443116 PMC2829758

[32] Cella D, Choi S, Garcia S, . Setting standards for severity of common symptoms in oncology using the PROMIS item banks and expert judgment. Qual Life Res. 2014;23(10):2651-2661. doi:10.1007/s11136-014-0732-6 24938431 PMC4710358

[33] Verbeke G, Molenberghs G, Verbeke G. Linear Mixed Models for Longitudinal Data. Springer; 1997.

[34] Naik UD. Some selection rules for comparing p processes with a standard. Commun Stat Theory Methods. 1975;4(6):519-535. doi:10.1080/03610927508827267

[35] Marcus R, Eric P, Gabriel KR. On closed testing procedures with special reference to ordered analysis of variance. Biometrika. 1976;63(3):655-660. doi:10.1093/biomet/63.3.655

[36] Lambert D. Zero-inflated Poisson regression, with an application to defects in manufacturing. Technometrics. 1992;34(1):1-14. doi:10.2307/1269547

[37] Rubin DB. Statistical Analysis With Missing Data. Wiley; 1987.

[38] White IR, Royston P, Wood AM. Multiple imputation using chained equations: issues and guidance for practice. Stat Med. 2011;30(4):377-399. doi:10.1002/sim.4067 21225900

[39] Douglas Bates M, Bolker B, Walker S. Fitting linear mixed-effects models using lme4. J Stat Softw. 2015;67(1):1-48. doi:10.18637/jss.v067.i01

[40] Brooks ME, Kristensen K, Van Benthem KJ, . glmmTMB balances speed and flexibility among packages for zero-inflated generalized linear mixed modeling. R J. 2017;9(2):378-400. doi:10.32614/RJ-2017-066

[41] Quartagno M, Grund S, Carpenter J. jomo: A flexible package for two-level joint modelling multiple imputation. R J. 2019;9(1):205-228. doi:10.32614/RJ-2019-028

[42] Pinheiro J. Linear and nonlinear mixed effects models. R package version. 2011. Accessed March 25, 2025. https://svn.r-project.org/R-packages/trunk/nlme/

[43] Whitehead L, Seaton P. The effectiveness of self-management mobile phone and tablet apps in long-term condition management: a systematic review. J Med Internet Res. 2016;18(5):e97. doi:10.2196/jmir.4883 27185295 PMC4886099

[44] Donkin L, Christensen H, Naismith SL, Neal B, Hickie IB, Glozier N. A systematic review of the impact of adherence on the effectiveness of e-therapies. J Med Internet Res. 2011;13(3):e52. doi:10.2196/jmir.1772 21821503 PMC3222162

[45] Kelders SM, Kok RN, Ossebaard HC, Van Gemert-Pijnen JE. Persuasive system design does matter: a systematic review of adherence to web-based interventions. J Med Internet Res. 2012;14(6):e152. doi:10.2196/jmir.2104 23151820 PMC3510730

[46] Sieverink F, Kelders SM, van Gemert-Pijnen JE. Clarifying the concept of adherence to eHealth technology: systematic review on when usage becomes adherence. J Med Internet Res. 2017;19(12):e402. doi:10.2196/jmir.8578 29212630 PMC5738543

[47] Beatty L, Binnion C. A systematic review of predictors of, and reasons for, adherence to online psychological interventions. Int J Behav Med. 2016;23(6):776-794. doi:10.1007/s12529-016-9556-9 26957109

[48] Barbera L, Sutradhar R, Seow H, . Impact of Standardized Edmonton Symptom Assessment System Use on emergency department visits and hospitalization: results of a population-based retrospective matched cohort analysis. JCO Oncol Pract. 2020;16(9):e958-e965. doi:10.1200/JOP.19.00660 32463762

[49] Aapro M, Bossi P, Dasari A, . Digital health for optimal supportive care in oncology: benefits, limits, and future perspectives. Support Care Cancer. 2020;28(10):4589-4612. doi:10.1007/s00520-020-05539-1 32533435 PMC7447627

[50] Grady PA, Gough LL. Self-management: a comprehensive approach to management of chronic conditions. Am J Public Health. 2014;104(8):e25-e31. doi:10.2105/AJPH.2014.302041 24922170 PMC4103232

[51] Howell D, Harth T, Brown J, Bennett C, Boyko S. Self-management education interventions for patients with cancer: a systematic review. Support Care Cancer. 2017;25(4):1323-1355. doi:10.1007/s00520-016-3500-z 28058570

[52] Bakouny Z, Paciotti M, Schmidt AL, Lipsitz SR, Choueiri TK, Trinh QD. Cancer screening tests and cancer diagnoses during the COVID-19 pandemic. JAMA Oncol. 2021;7(3):458-460. doi:10.1001/jamaoncol.2020.7600 33443549 PMC7809614

[53] Perry LM, Peipert JD, Kircher SM, Cantoral J, Penedo FJ, Garcia SF. Adverse COVID-19 experiences and health-related quality of life in cancer survivors: indirect effects of COVID-19–related depression and financial burden. J Patient Rep Outcomes. 2023;7(1):71. doi:10.1186/s41687-023-00601-y 37458820 PMC10352476

[54] Yu L, Liu YC, Cornelius SL, . Telehealth use following COVID-19 within patient-sharing physician networks at a rural comprehensive cancer center: cross-sectional analysis. JMIR Cancer. 2023;9:e42334. doi:10.2196/42334 36595737 PMC9848440

[55] Momenimovahed Z, Salehiniya H, Hadavandsiri F, Allahqoli L, Günther V, Alkatout I. Psychological distress among cancer patients during COVID-19 pandemic in the world: a systematic review. Front Psychol. 2021;12:682154. doi:10.3389/fpsyg.2021.682154 34650469 PMC8506116

