# Supplementary material for: Web-Based Cancer Symptom Self-Management System: A Randomized Clinical Trial
Source: JAMA Netw Open. 2025 May 5;8(5):e258353. doi: 10.1001/jamanetworkopen.2025.8353 (PMC12053558; doi:10.1001/jamanetworkopen.2025.8353)
Supplement: Supplement 1. — Trial Protocol and Statistical Analysis Plan [file jamanetwopen-e258353-s001.pdf]

PROTOCOL TITLE:

Implementation and Evaluation of an Expanded Bilingual Electronic Symptom Management Program across a Multi-site, Fully-integrated Comprehensive Cancer Center (NU IMPACT)

PRINCIPAL INVESTIGATOR:

Name: David Cella, PhD Department: Medical Social Sciences

CO-INVESTIGATORS:

Name: Mike Bass, MS
Department: Medical Social Sciences

Name: Sofia Garcia, PhD Department: Medical Social Sciences

Name: Michael Kallen, PhD Department: Medical Social Sciences

Name: Sheetal Kircher, MD
Department: Northwestern Medicine Hematology Oncology

Name: Frank Penedo, PhD Department: Medical Social Sciences

Name: Denise Scholtens, PhD Department: Preventive Medicine

Name: JD Smith, PhD Department: Psychiatry

Name: Betina Yanez, PhD Department: Medical Social Sciences

Name: Christopher George, MD
Department: Northwestern Medicine Hematology Oncology

Name: Dean Tsawhras, MD
Department: Northwestern Medicine Hematology Oncology

Name: Ann Marie Flores, PT, MSPT, PhD, CLT
Department: Physical Therapy and Human Movement Sciences

Name: Kimberly A. Webster, MA
Department: Medical Social Sciences

VERSION DATE: 9/13/2024 (version 15.7)

1.0 Purpose of the study:

We will implement and evaluate a fully electronic health record (EHR)-integrated oncology symptom assessment and management program across NMHC, on patient- and system-level outcomes, for patients receiving treatment with curative intent, non- curative intent or post-treatment survivorship care.

NU-IMPACT Project Aims

This study will focus on **Aim 2** as described in the grant**:** ***Evaluate the impact of the program by conducting a hybrid Type I/II effectiveness-implementation trial to evaluate the impact of the enhanced care program (EC: symptom monitoring & self-management intervention), relative to usual care (UC) on patient- and system-level outcomes*****.**

In the second phase of the study (starting at post-implementation), we will include **Aim 3** as described in the grant: ***Identify facilitators and barriers to implementation and disseminate to other health systems***

We will test the effectiveness of a health care system-wide symptom management intervention, implemented across eight adult hematology/oncology, gynecologic oncology and radiation oncology outpatient clinics at Northwestern Memorial HealthCare Corporation (NMHC). A customized PRO assessment portal, NMPRO, built to work within Epic's MyChart portal and its hyperspace, delivers assessments from the Patient Reported Outcomes Measurement Information System® (PROMIS®), and includes options for custom assessments. This enables routine symptom assessment across the NMHC network, including the eight oncology outpatient clinical practices in this study. Using a clinic-level randomized roll-out implementation trial design, we will further test, using an embedded patient-level randomized controlled clinical trial, the effectiveness of an enhanced care (EC) approach to actively engage and motivate participants, in monitoring and managing their symptoms. The existing NMPRO will constitute usual care (UC), and added patient engagement and activation features, via a tailored, patient web-based tool, will comprise the EC condition. This innovative Type I/II hybrid trial design allows for a within- and between-site evaluation of implementation along with a sufficiently powered group-based comparison to demonstrate effectiveness on individual patient outcomes.

Diversity Supplement and Aims (Grant # 3UM1CA233035-01S1):

The parent grant (NU IMPACT) has utilized the cPRO symptom monitoring questionnaire that prospectively monitors physical functioning severity with the Patient Reported Outcome Measurement Information System - Physical Function (PROMIS-PF) survey via cPRO.

This sub-study will allow us to: 1) leverage the parent grant to map the development of PFs across different cancer patient populations; 2) develop a learning health systems mechanism to support oncology providers to match the right patient, at the right time, with the right rehabilitation service, in the right location (4Rs Referral System); and 3) obtain stakeholder feedback about implementation and dissemination strategies for multi-level (patient, provider, community, system) prevention, detection, and management of cancer-related PFs and the development of risk stratification models for rehabilitation referral. The results from this supplement will inform

the planned R01 resubmission to the NCI to test the combination of cPRO + 4Rs + pre-hab in a large-scale randomized controlled trial.

Please note: Since the sub-study builds off of the main study. The sub-study will not recruit additional patients for analysis, instead it will utilize the consented patients for analysis.

Supplement Aims:

Supplement Aim 1: ***Map the trajectory and identify risk factors for development of physical and functional impairment and for cancer rehabilitation services utilization from the point of cancer diagnosis through at least the first year after diagnosis.*** We hypothesize that cancer patients with different tumor types and treatment modalities will differ in their physical function scores from PROMIS® (PROMIS-PF) and utilization of cancer rehabilitation services (physical, occupational, and speech therapies - PT, OT, ST, respectively) across time. We will also identify the time of first onset of CTCAE Grade 2 adverse events that warrant consideration of rehabilitation (e.g., lymphedema, fatigue, pain, peripheral neuropathy). We will achieve this aim with data prospectively gathered from the parent study and our institution's Electronic Data Warehouse (EDW) curation of medical and demographic data from our system's fully-integrated, customized electronic health record (Epic) to identify risk factors for development of PFEs.

Supplement Aim 2: ***Modify NMPRO to provide a decisional support mechanism for early and timely cancer rehabilitation referral in response to electronic health record alerts generated in the parent study.*** We hypothesize that providing 4Rs decisional support to oncology providers treating patients in the EC arm will result in more and early ("pre-hab") referrals and, in turn, increase pre-hab utilization when compared to patients in the UC arm of the parent study. To achieve this aim, Dr. Flores will work with Dr. Cella and his NUIMPACT team to modify NMPRO in the EC arm to integrate the 4Rs decisional support with the EPIC interface through "best practice alerts" (BPAs). The BPAs will match the right patients (those PROMIS-PF scores normal that trigger an alert to the oncology provider), to the right rehabilitation service (PT, OT, ST), at the right time (first alert), in the right place (patient and rehabilitation clinic matched by zip code). Estimates from this aim (referral rates, changes in PROMIS-PF scores, type of rehabilitation service) will help our team identify what is working well and what needs to be modified for use in our planned R01 resubmission.

Since cPRO is already embedded in Epic and results are viewed by our oncology providers, we have uncovered a gap in care regarding physical and functional decline that needs to be addressed. We have partnered with Dr. Ann Marie Flores and received additional funding from the NCI to address this gap. Dr. Flores' research shows that the single biggest barrier to care that can address physical and functional impairment is lack of referral for cancer rehabilitation.

We have developed a clinical decisional support system that will match the right patient, at the right time, with the right rehabilitation service in the right place (4Rs). This system will incorporate a best practice alert that will be triggered in response to PROMIS-PF scores and leverage our vast NM Outpatient Rehabilitation Services clinic network.

Supplement Aim 3***: Identify realistic implementation, dissemination, and adaptation strategies for future hybrid effectiveness trials of pre-habilitation interventions for different cancer patient populations.*** We will conduct focus groups with key stakeholders (patients, oncology providers,

rehabilitation specialists [PT, OT, ST], clinical administrators, patient service representatives) to learn how to implement, disseminate, and adapt the 4Rs to include risk stratification models, respond to changes in patient and provider behaviors, and different clinical settings (inpatient v. outpatient; academic v. community). This early planning is recommended by the NCI.

2.0 Background / Literature Review / Rationale for the study:

Our Research Center will implement a rigorous EHR-integrated symptom monitoring and management system. Our preliminary data suggest that this will improve symptom control and associated quality of life, healthcare utilization, and cancer treatment delivery. This project addresses a very important problem faced by patients with cancer at all phases of treatment: Symptoms that can be identified and treated early, thus producing better clinical, treatment utilization, and humanistic (patient-reported) outcomes. We approach this problem by addressing two critical barriers to progress. One barrier, the health care delivery system itself, will be addressed by introducing a patient-centered learning health system that pivots on assessment and early management of symptoms. Another barrier, the need of many patients to actively participate in their care, will be addressed with tailored, relevant self-assessment and self-management tools for living a healthy life with cancer. Our success in this project can have local and even national effects.

At the local level, we anticipate that the intervention will help patients obtain better symptom control, and will help the clinical delivery system identify and improve its practices and culture in the service of better care for future patients (i.e., we anticipate true adoption). At the national level, based on our leadership in areas of patient- centered supportive care and patient-reported outcomes, and upon our anticipated results, we expect that there will be other comprehensive cancer centers and regional cancer care programs who seek to adopt the supportive care methods, technologies, and treatments we successfully implement at NMHC. Additionally, with our participation in the IMPACT Consortium, we believe that we in turn will learn from the successes of others in this era of value-based, patient-centered cancer care delivery. The IMPACT project is one of several initiatives funded by the National Cancer Institute (NCI) Cancer Moonshot that are aimed at accelerating a decade's worth of cancer research in five years. As a cooperative grant, three Research Centers and a Coordinating Center work collaboratively to conduct evidence-based research to improve symptom control for cancer patients. IMPACT was designed to accelerate the use of effective symptom management systems that collect patient-reported data and support clinical responses consistent with evidence-based guidelines. As such, each Research Center is implementing their own ePRO interventions. Using implementation science approaches, IMPACT will evaluate the adoption of integrated electronic systems for monitoring and managing patient-reported symptoms in routine cancer care.

In support of the sub-study, physical and functional symptom burden is known to be high among cancer survivors and is an ideal target for chronic care management. Cancer-related physical and functional side effects (PFs) include joint pain, fatigue, muscle weakness, loss of range of motion, altered skin sensation and integrity, postural abnormality, and lymphedema. Cancer survivors lack the knowledge and skills to prevent, detect, and manage PF side effects. Our research shows that a majority of cancer survivors report 3 or more long-lasting PFs and that rehabilitation services are

under-utilized to treat them. Cancer rehabilitation interventions can reduce PFs and support functional ability. However, our work shows that cancer survivors lack receptivity to and misunderstand the benefits of cancer rehabilitation – even among those with cancer-related disability. Oncology providers also lack system support for timely identification of PFs and referral to rehabilitation. Usual cancer care lacks ownership for effective surveillance and management of PFs that can be done without interrupting clinical flow and overburdening providers and patients. Early rehabilitation (pre-hab) could facilitate ownership of PFs by educating patients on early detection and self-management of PFs, but this approach is completely absent from usual cancer care. The parent grant (NU IMPACT) has developed the cPRO symptom monitoring questionnaire that prospectively monitors PFs severity with the Patient Reported Outcome Measurement Information System - Physical Function (PROMIS-PF) survey.

With this sub-study, we will be able to: 1) leverage the parent grant to map the development of PFs across different cancer patient populations; 2) develop a learning health systems mechanism to support oncology providers to match the right patient, at the right time, with the right rehabilitation service, in the right location (4Rs Referral System); and 3) obtain stakeholder feedback about implementation and dissemination strategies for multi-level (patient, provider, community, system) prevention, detection, and management of cancer-related PFs and the development of risk stratification models for rehabilitation referral. The results from this supplement will inform the planned R01 resubmission to the NCI to test the combination of cPRO + 4Rs + pre-hab in a large-scale randomized controlled trial.

3.0 Inclusion and Exclusion Criteria:

Aim 2

Participants will represent three phases of the cancer treatment continuum:

1. Treatment with curative intent (i.e., as patients receive chemotherapy or biological therapy with curative intent);
2. Treatment with non-curative/palliative intent, including patients with advanced or metastatic disease who receive chemotherapy or biological therapy to control or slow the advancing of their disease, thereby relieving symptoms and/or extending progression-free survival and overall survival; and
3. Cancer survivorship (i.e., patients who have completed active cancer treatment with curative intent and are free of any evidence of disease, including those who are receiving maintenance or prophylactic hormonal or other cancer treatment.

INCLUSION CRITERIA:

1. Curative Intent Group:
    - ≥ 18 years of age;
    - medical chart confirmed diagnosis of a solid or hematological malignancy within the last 10 years;
    - had an appointment at a participating site with a participating oncologist in the last year;
    - willingness to be randomized (post-implementation only);
    - have initiated primary treatment with curative intent within the past 3 months (i.e., as patients receive surgery, standard- or high-dose chemotherapy, biological therapy, and/or radiotherapy);

- provider confirmed planned treatment and follow-up within the Northwestern Medicine hospital and clinics;
- provider confirmed planned treatment and follow-up within the Northwestern Medicine hospital and clinics; and
- able to read English or Spanish.

2. <u>Non-Curative/Palliative Intent Group:</u>
   - ≥ 18 years of age;
   - medical chart confirmed diagnosis of advanced or metastatic solid or hematological malignancy within the last 10 years;
   - had an appointment at a participating site with a participating oncologist in the last year
   - provider confirmed planned treatment and follow-up within the Northwestern Medicine hospital and clinics;
   - willingness to be randomized (post-implementation only);
   - undergoing cancer treatment with non-curative/palliative intent (e.g., patients with advanced or metastatic disease who receive chemotherapy, biological therapy, and/or radiotherapy to control or slow advance of their disease);
   - provider confirmed planned treatment and follow-up within the Northwestern Medicine hospital and clinics; and
   - able to read English or Spanish.
3. <u>Cancer Survivorship Group:</u>
   - ≥ 18 years of age;
   - medical chart confirmed diagnosis of solid or hematological malignancy within the past 10 years;
   - had an appointment at a participating site with a participating oncologist in the last year;
   - provider confirmed planned treatment and follow-up within the Northwestern Medicine hospital and clinics;
   - willingness to be randomized (post-implementation only);
   - completed active cancer treatment with curative intent, including those who may be receiving maintenance or prophylactic cancer treatment;
   - provider confirmed planned cancer follow-up care (with or without treatment) within the Northwestern Medicine hospital and clinics; and
   - able to read English or Spanish.

<u>Aim 3</u>

- *Clinician Focus Groups*
  - Clinical or operational leader in an NM clinic participating in cPRO implementation
  - Familiarity with/experience using cPRO for at least 1 year
- *Patient-facing Interviews*

  We plan to conduct individual patient interviews with participants from three different cohorts:
  - **Cohort 1: Enhanced Care User Group**:
    - Met eligibility criteria for parent study (listed in Inclusion and Exclusion Criteria for Aim 2)
    - Completed NU IMPACT post-implementation study in past 3 months
    - Randomized to the Enhanced Care group in the parent study

    - Did not withdraw from the parent study
    - Accessed the MyNM Care corner at least once during study participation
    - English or Spanish speaking

- **Cohort 2: Enhanced Care non-User Group**
    - Met eligibility criteria for parent study (listed in Inclusion and Exclusion Criteria for Aim 2)
    - Completed NU IMPACT post-implementation study in past 3 months
    - Randomized to the Enhanced Care group in the parent study
    - Did not withdraw from the parent study
    - Never accessed the MyNM Care corner during study participation
    - English or Spanish speaking

- Cohort 3: **Non-Consented**
    - Met eligibility criteria for parent study (listed in Inclusion and Exclusion Criteria for Aim 2)
    - Electronic medical record designation of Hispanic/Latino ethnicity and preferred Spanish language
    - Invited to participate in the NU IMPACT post-implementation study but did not consent

<u>Supplement Aim 3:</u>

- *Clinician Focus Groups*
    - Clinical stakeholders include clinicians and/or clinical staff members who have used the 4Rs system in any capacity for the purposes of sending rehabilitation referrals during the pilot.
- *Patient Focus Groups*
    - Consented under parent study
        - Patients enrolled in the parent study (NU IMPACT) and who received a referral to cancer rehabilitation services (PT, OT, ST, and/or PM&R) as a result of their provider having used the 4Rs system during the Supplement pilot.
    - Non-consented under parent study
        - If non-consented patients received a referrel to cancer rehabilitation services (PT, OT, ST, and/or PM&R) as a result of the 4Rs system being used as standard clinical workflow, these patients will also be eligible to participate in patient focus groups.
        -

4.0 Sample Size:

For **Aim 2**, this study will involve voluntary participation of an estimated 12,671 English- and Spanish-speaking participants, with the estimate based upon a 70% acceptance rate and the 2016-

2017 patient volume statistics. Eligibility includes patients receiving treatment with curative intent, treatment with palliative (non-curative) intent, and disease-free survivors. Based on the demographic characteristics of ambulatory oncology patients across six of the eight target clinics (radiation oncology estimates are not included), we anticipate that 13% of our participants will be Hispanic/Latino (hereinafter referred to as Hispanic), 13.3% African American, 4% Asian, and about 69.7% non-Hispanic white. We anticipate that our sex distribution will be about 45% male and 55% female, and that about 40% of our Hispanic participants will either be Spanish monolingual or endorse Spanish as a language of preference.

Based upon 2016-2017 experience, we project an annual volume of over 18,000 unique patients across the six of the eight clinical units, ranging from 1,500-4,500 per unit. We will study implementation of the NMPRO system, including an enhanced care (EC) component as part of a randomized trial, for all adult patients, being treated in all three phases of the cancer treatment continuum. Although there is some cross-unit variability in proportion of patients per treatment phase, we estimate that on average each unit will see 40% of its new patients being treated for cure, 30% will be treated for life extension, and 30% will be disease-free survivors. Based upon our prior experience with very similar studies, we anticipate conservatively that 70% of patients will agree to participate in the study. We also have extensive experience in retention of patients in studies with follow-up out to 1-2 years. Therefore, we anticipate 10% dropout (20% in palliative setting) for this study. Assuming a nine-month enrollment at each site, this will produce the anticipated sample size.

We plan to enroll **12,671 participants** (across 8 clinical sites) for the Type I/II hybrid trial that consists of pre-implementation cohort and post-implementation, randomized cohort to evaluate the implementation of the intervention. For the pre- implementation/non-randomized cohort, we aim to enroll 6,336 participants who will complete additional REDCap ePROs along with the PROMIS CATs and supportive care needs assessment that are part of NMPRO. Participants will be stratified by region, clinical site and cancer treatment continuum phase. Additionally, within each region, intervention roll-out to clinical sites will be randomized.

For the post-implementation randomized trial cohort, again, we aim to enroll 6,336 participants who will be randomized into the usual care (UC) arm or the enhanced care (EC) arm to test the effectiveness of a system-wide symptom management intervention. As in the pre-implementation, the UC arm will complete additional REDCap ePROs along with the PROMIS CATs and supportive care needs survey, called "*Your pre-appointment cancer symptom and needs assessment (cPRO-Monitor)*"questionnaire, that are part of NMPRO, which constitutes usual care. The EC arm will also complete additional REDCap ePROs along with the PROMIS CATs and supportive care needs assessment that are part of NMPRO, as well as receive patient engagement and activation features via a *patient-facing, tailored, web-based tool*. The web-based tool will be called My NM Care Corner. Within each trial arm (approximately 3,168 participants per arm), participants will be stratified by region, clinical site and cancer treatment continuum group.

For our qualitative work in **Aim 3**, we plan to enroll up to 30 clinicians, 10 administrators and 50 patients to explore facilitators and barriers to the implementation of cPRO and patient experiences with the intervention. Each clinician focus group will include 6-12 participants, generally regarded as sufficient for facilitating both individual contributions and group dynamics in a controlled

setting. For patient-facing individual interviews, we plan to recruit 15 patients each across the three cohorts.

For aim 3 of the supplement study, both the clinician and patient facing focus groups will include 6-10 participants.

5.0 Research Locations:

Research will be conducted at oncology clinical settings in the four Northwestern Medicine regions: Central (Chicago metropolitan area), West (western Chicago suburbs) North (northeastern Illinois), and South (south and southwest Chicago suburbs). All required permission and/or approvals will have been obtained at each clinical setting prior to project implementation.

For Aim 2, participants will complete the *"Your pre-appointment cancer symptom and needs assessment (cPRO-Monitor)"* (through Epic MyChart portal) as part of standard care and the study REDCap assessments via the web or a smartphone from the comfort of their homes, or via tablets or computers at cancer clinic offices.

Additionally, for the EC cohort only, once participants complete the current NMPRO symptom monitoring, they will receive tailored, web-based, patient-centered information based on their symptom severity. The web-based tool will contain information, so that the participant can gain knowledge on cancer-related outcomes, improve self-management skills, improve communication skills, and empower them to improve motivation via the web or a smartphone from the convenience of their homes.

6.0 Multiple sites:

This project will be conducted across **eight NMHC oncology clinical units** that collectively provide oncology services to over 18,000 unique patients yearly (ranging from 1,500- 4,500 patients per unit). Those NMHC oncology clinical units are presented in Table 1 below.

Table 1. NMHC Regions, Clinical Units, Clinical and Administrative Leads, and Annual Patient Volume

NMHC Regions	Clinical Unit	Clinical Lead	Admin Lead
Central	Med/General Hematology /Oncology	Kircher	Caires
Central	Women's Cancer (Gynecologic Oncology & Breast Medical Oncology)	Kircher	Caires
Central	Olson (Development Therapeutics)	Kircher	TBD
North	Medical Oncology & Gynecologic Oncology	Tsarwhas	Wilson-Kramer
West	Delnor Cancer Center Hematology / Oncololgy	George	Smiley
West	Warrenville Cancer Center Hematology / Oncology	George	Smiley
West	Kishwaukee Cancer Center Hematology / Oncology & Regional Medical Group Gynecologic Oncology	George	Smiley
South	Orland Park Cancer Center	George	Frey

All sites will follow the same procedures as described in section 8.0 of this protocol.

7.0 Reliance Agreements/Single IRB:

The study team has executed a Reliance agreements with participating sites in the NM West Region. Northwestern IRB will be the oversight IRB for all participating sites. Documentation of executed reliance agreements have been provided to and approved by NU IRB.

8.0 Procedures Involved:

As stated above, we will test the effectiveness of a system-wide symptom management intervention, implemented across eight adult hematology/oncology, gynecologic oncology outpatient and radiation oncology clinics at Northwestern Memorial HealthCare Corporation (NMHC). The system-wide symptom management intervention will augment the customized PRO assessment portal, called NMPRO, built to work within Epic's MyChart portal and its hyperspace, delivers assessments from the Patient- Reported Outcomes Measurement Information System® (PROMIS®), and includes options for custom assessments. This enables routine symptom assessment across the NMHC network, including the eight oncology outpatient clinical practices in this study.

Using a clinic-level randomized roll-out implementation trial design, we will further test, using an embedded patient-level randomized controlled clinical trial, the effectiveness of an enhanced care (EC) approach to actively engage and motivate participants, in monitoring and managing their symptoms. The existing NMPRO will constitute usual care (UC), and added patient engagement and activation features via a tailored, patient- facing, web-based tool will comprise the EC condition. Participants will be enrolled into the Pre-Implementation or Randomized cohorts (See Figure C.7 below)

Figure C.7. Participant Flow by Implementation and Randomization Condition

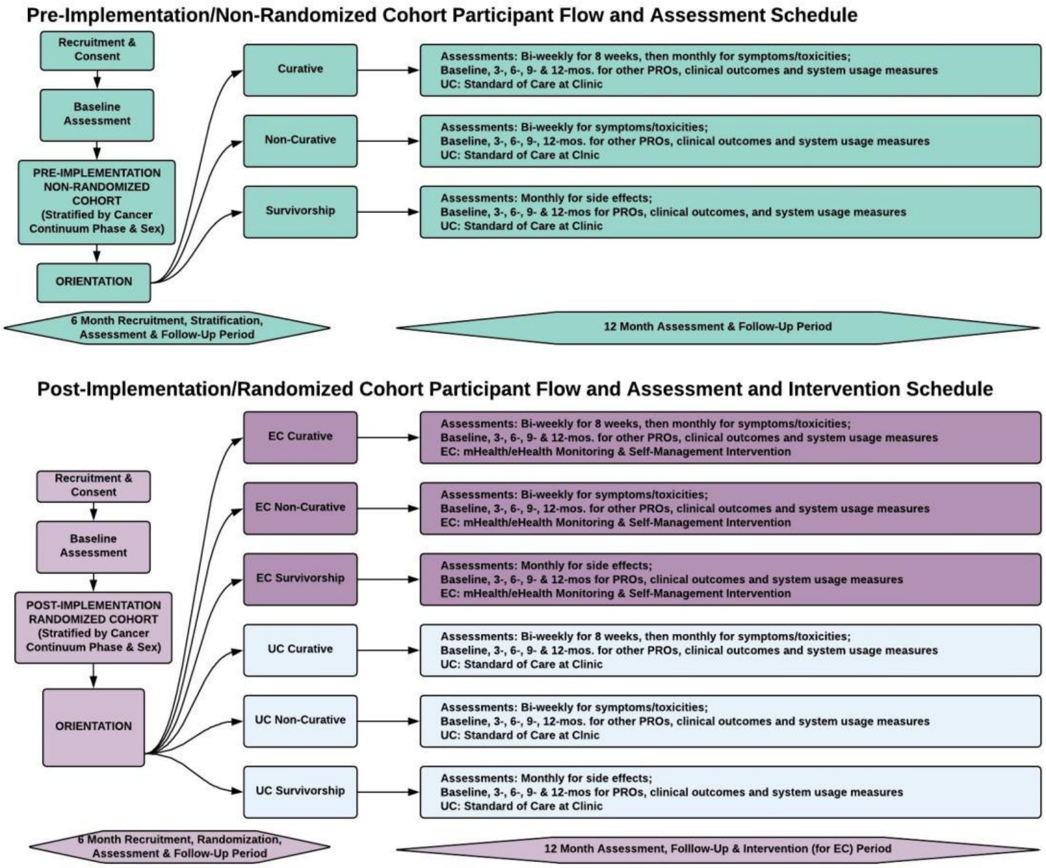

All participants in the pre-implementation or randomized cohorts will undergo ongoing symptom and problem checklist monitoring, i.e., *Your pre-appointment cancer symptom and needs assessment (cPRO-Monitor)*, with real-time I integrated alerts over a 12-month period. To reduce patient burden, the frequency of study monitoring will be monthly for all cancer continuum phase groups.

The comprehensive REDCap ePRO assessments and symptom monitoring can be completed via the web or a smartphone from the comfort of their homes, or via tablets or computers at cancer clinic offices. The comprehensive REDCap ePRO assessments take 25 minutes on average to complete, while the Symptom and Needs Assessment questionnaire take 6-7 minutes. Table 2 displays the assessment frequency, time required for participation at each time point, and measures completed. Data will be collected by leveraging cancer clinics' existing approach to symptom monitoring via Northwestern Medicine Patient Reported Outcomes (NMPRO) infrastructure and the REDCap built-in standardized measures (outlined in Table 2). Participants in both cohorts Pre-I and randomized: UC vs. EC) will complete the same activities.

Table 2 REDCap Study Assessment Frequency

Measure Assessment Battery		12-month Enrollment Period											
	BL	1	2	3	4	5	6	7	8	9	10	11	12
REDCap Symptom Monitoring (PROMIS: Pain, Fatigue, Anxiety, Depresion, & Physical Function; PRO-CTCAE; & Godin) [10-12 minutes]	x	X	x	x	x	x	x	x	x	x	x	x	x
REDCap Comprehensive Assessment (Demographics (i.e., education, employment, insurance and captured only at baseline); CAHPS; PROMIS: Self-efficacy Managing Symptoms, Self- efficacy Managing Medication and Treatment, Informational, & Instrumental; CASE; FACT-G7; SILS; 1-item COST-FACIT; CollaboRATE) [25 -35 minutes]	x			x			x			x			x
REDCap Resource Utilization [2-3 minutes]	x						x						x

Pre-Implementation Phase:

As part of NM oncology standard of care, NMPRO system sends out the *"Your Pre-appointment Symptom and Needs Assessment (cPRO-Monitor)"* questionnaire to patients 72 hours prior scheduled oncology visit via MyChart. At the start of the study, the NMPRO Symptom and Needs Assessment questionnaire originally consisted of PROMIS Depression, Anxiety, Fatigue, Pain Interference and Physical Function CATs, along with a supportive care checklist in which patients endorse any supportive care needs for which they want to be contacted by a health care provider. However, as part of an ongoing QI initiative to implement and evaluate NMPRO in parallel to this protocol, workflow limitations that were encountered after we began implementation required that we shorten the PROMIS assessments to just two items per symptom domain. The two-item short forms are comprised of a subset of items from the longer, already-approved CAT approach. If the patient does not complete the NMPRO Symptom and Needs Assessment questionnaire prior to scheduled oncology visit, they will have the option to complete the NMPRO Symptom and Needs Assessment in clinic. NMRPRO alerts generated by elevated scores on the Symptom and Needs Assessment questionnaire are responded by social work (for anxiety, depression or practical needs) or the medical team (for fatigue and physical function) or dietitian (for nutritional needs) within 72 hours. Participants are clearly instructed that in case of an emergency or the need for immediate care, patients should call 911, visit the ER or the urgent oncology care clinics.

For the pre-implementation phase, patients that consent to be in the study will receive monthly REDCap ePRO assessments for a 12-month period. Participant will have the option to complete REDCap consent and questionnaires in English or Spanish. All patient-facing study materials were translated to Spanish (and submitted to the IRB). Once enrolled in the study, the monthly symptom assessment (of physical function, pain interference, depression, anxiety, fatigue, nausea, vomiting, constipation, shortness of breath, diarrhea, physical activity, and insomnia). Participants will be stratified by clinical unit based on the size of each clinical unit. Additionally, participant that complete the REDCap assessment will be entered into a raffle for $100 Amazon gift card for each quarter of 12-month enrollment period. The expanded REDCap Comprehensive assessments are expected to take 25-35 minutes at each time point. If a participant wishes to participate and does not have a computer/tablet or smartphone with internet connection or a disability that would make it difficult for them to complete a survey using a computer/tablet or smartphone, they may complete the survey by telephone interview with a trained research assistant. Those who need to complete the survey by phone are instructed to contact the study coordinator to schedule a time. Consented participants who do not start or only partially complete the full REDCap assessment for each time point will receive automated REDCap email reminders every two days for up to two times. If not completed during this time, a study research assistant will call two times to remind participants to complete the REDCap assessment and offer assistance if needed to complete the assessment.

Measures. As detailed above, the NMPRO Symptom and Needs Assessment questionnaire consists of PROMIS Depression, Anxiety, Fatigue, Pain Interference and Physical Function CATs, which were later shortened from this approach to a subset of just two items per domain, along with a supportive care checklist in which patients endorse any supportive care needs for which they want to be contacted by a health care provider. This checklist includes various areas of potential distress (e.g. financial, physical, spiritual), and patients are asked to indicate their areas of distress. The assessment is standard of care and takes approximately 6-7 minutes to complete.

The additional REDCap PRO measures completed by participants for **Aim 2** will consist of:

1. The Functional Assessment of Cancer Therapy (FACT) G7 measure
2. Composite scales of the Cancer CAHPS questionnaires
3. Resource Utilization,
4. a short series of patient-reported health care/resource utilization questions
5. Select items from the PRO-CTCAE measure (nausea, vomiting, constipation, shortness of breath, diarrhea, and insomnia)
6. A single item regarding financial toxicity of cancer care
7. Single item of the health literacy questionnaire (SILS)
8. Sociodemographic questions
9. A version of t13ollaborateATE measure
10. PROM–S - Self-Efficacy for Managing Medications and Treatments
11. PROMIS – Self-Efficacy for Managing Symptoms
12. PROMIS v2–0 - Informational Support

13. PROMIS v2–0 - Instrumental Support
14. CA–E - Seek and Obtain Information subscale
15. Godin physical activity questionnaire
16. Patient-reported demographics, i.e., education, insurance type, employment status, gender identity
17. PROMIS Symptom Measures that collectively constitute the Cancer Center Symptom and Needs Assessment
    - PROMIS Bank v1.2- Physical Function
    - PROMIS BANK v1.0 – Anxiety
    - PROMIS Bank v1.0 – Depression
    - PROMIS Bank v1.0 – Fatigue
    - PROMIS Bank v1.1 – Pain Interference

Documented health care service utilization will be assessed in **Aim 2** using a limited dataset of EDW queries EHR data.

Consented Patients:

Data from REDCap will be linked to utilization/clinical data from the EDW through an 'honest broker' (NM EDW analyst) who will link study data from REDCap to data from the EDW. The data will be considered a limited dataset because zip code will be included for analyses related to social determinants of health. The EDW analyst will automate exports stripped of sensitive protected health information (e.g., encounters related to substance abuse or providers related to a psychiatric specialty) and any identifiable information, except zip code. Data exports will be generated quarterly and will be saved to a shared folder on Northwestern's secure FSMresfiles system with access restricted to NU IMPACT study personnel. Once EDW data has been extracted and shared with study team, there will be no efforts to re-identify data. Previous studies led by Drs. Garcia and Penedo have demonstrated their ability to pull reliable and comprehensive health service usage data from NM's EHRs across all geographic regions. They have developed a query that includes date (for this project dates will be converted to days from date of consent) and type of health care contacts, clinic/department, and provider. Data from all eligible records can be pulled from EHRs and NM Tumor Registry by the EDW and delivered to study statisticians as a limited data set. Working with oncology clinicians, the research team developed a coding schema to summarize the available health usage data. Major categories include in-person (medical visit) and telephone contacts and MyChart communications and usage; sub-categories include relevant departments (Medical, Surgical, Radiation and Supportive Oncology; Palliative and Emergency Care; Integrative Medicine; Other specialty, such as, referral encounters related to rehabilitation (PT, OT, Speech); Internal Medicine; Lab services). In-person contacts have an additional classification for inpatient or outpatient encounters. Usage will be summarized as the number of encounters within and across categories within set timeframes. Additionally, confounding factors will be extracted: sex, age, zip code, cancer type and stage, treatment type, time since diagnosis, time since last treatment, comorbidities, race/ethnicity, and language preference.

Non-Consented Patients:
A data use agreement established between Northwestern Medicine and Northwestern University was amended in July 2023 to facilitate the transfer of EDW data for this study's non-consented patient cohort in a way that complied with Illinois state privacy law. This amended data use agreement outlined safeguards and appropriate use of such data, including zip codes and sensitive protected health information. In August 2024, the Northwestern Medicine Privacy Steward advised the study team that a recent amendment to Illinois state privacy law has negated the need for the 2023 data use agreement, and that a HIPAA Waiver of Authorization will now cover the release of protected information, most significantly zip code and sensitive protected health information. Per IRB guidance, the study team has requested waivers of consent and HIPAA authorization for this study cohort.

In **Aim 2**, data will also collect *patient-reported health care service usage* using a selection of Resource Usage questions as part of the REDCap study assessment. These questions will assess the quantity of various health care services outside of the NM network (visits inside the NM network will be captured via EDW query), including the following services: physician visits (primary and specialty care, psychologists, psychiatrists); emergency department visits; inpatient hospital services. The health care usage questions will be administered in at baseline, 6 and 12 months. Additionally, *patient-reported health care satisfaction* will also be collected using Cancer CAHPS items at baseline, 3, 6, 9, and 12 months.

We have found that regular contact with participants (e.g., reminder emails) improves retention rates. Therefore, we will use strategies we have implemented in prior studies to maximize participant engagement and retention (e.g., reminder emails and/or phone calls, mailing study updates, etc.). As stated above, for each REDCap assessment completed, a participant will be enrolled in a raffle for a $100.00 Amazon gift card. That is, if a participant completes the baseline assessment and the 3-month assessment, then the participant's name will be included in the raffle pool twice. During the participant enrollment phase, there will be one raffle drawing each quarter (from the start date of participant enrollment) for all regions until the end of the project's participant data collection phase. Only prospective assessments during the quarter will be eligible to be included in that quarter's participant raffle. There will be five winners per quarter.

Post-Implementation Phase:
Aim 2. In post-implementation, the randomized control intervention will be implemented and will follow the same protocol as pre-implementation. However, participants will be randomized into the usual care (UC) arm or the enhanced care (EC) arm. The UC arm will follow the same protocol as stated above with no deviation. The EC arm will have additional self-management intervention. Participants randomized into the EC arm will receive web-based, patient-centered information via My NM Care Corner once consented via text message or email (provided in both English and Spanish) to:

- Gain knowledge on cancer-related concerns
- Improve Self-management skills
- Improve communication skills
- Empower them to improve motivation and self-management

During the time of participation in the study, if the NMPRO *"Your pre-appointment cancer symptom and needs assessment (cPRO-Monitor"* questionnaire is completed, the EC participant will be sent a study reminder email or text message via REDCap (based on a nightly EDW pull of NMPRO scores of EC participants) providing information on how to access patient materials with a secure URL link to the EC condition website (i.e., My NM Care Corner) available in English and Spanish and will be able to it access at any time. The Spanish version of the will be available to participants whose preferred language is Spanish and consented with the Spanish version of the consent form.

Patient-facing materials will be targeted to common symptoms related to their cancer experience. Furthermore, there will be an audio option available in English and Spanish for all material presented on the website and a patient-video (again, English and Spanish versions) will be on the homepage to educate participants on type information available and navigation options. Participants will receive helpful information on NMPRO responses. For example, if a participant endorses moderate to severe anxiety during a NMPRO assessment, the EC condition website (i.e., My NM Care Corner) will release information on the management of anxiety to the participant's website dashboard. Participants will be able to navigate to other helpful information (e.g., symptom library and patient resources) besides information presented in their dashboard. Participants with normal values for all domains will also receive the link and will be able to navigate to information to help manage symptoms that they may be concerned about, even though they did not endorse the symptom via a NMPRO assessment.

Patients will receive automated reinforcement emails, calls or text messages encouraging them to access the EC condition website (i.e., My NM Care Corner) and content based on participant's NMPRO results. We will track activity on the website for analysis purposes regarding usability and outcomes. The EC condition website will be developed by and hosted at FSM and will not contain PHI. These links to the website will contain an encrypted study identifier and an encrypted list of the participant's symptoms as collected through NMPRO. See links below:

English: http://symon.fsm.northwestern.edu/impact/en/
Spanish: http://symon.fsm.northwestern.edu/impact/es/

When a participant clicks on the link, the symptom management website will track that the encrypted participant ID initiated a session at a given date and time, and will then track all web pages viewed within that session. In addition, a scheduled process from an FSM-hosted server will be used to generate SMS/text links identical to those available within MyChart NMPRO results page (see below), which will ultimately be distributed by a 3rd party (Twilio).

EC participants will be able to review their NMPRO *"Your Pre-appointment Symptom and Needs Assessment (cPRO-Monitor)"* results in MyChart. Epic will identify EC participants based on the project's StudyTracker's study arm: Enhanced Care, which will trigger Epic to release the My Chart results to only EC participants. Within the MyChart Personalized Questionnaires tab, there will be a graphical representation of symptom scores over time and score summary table, so that EC participants can see how they are doing over time and a link to the EC condition website (identical to those sent via REDcap), My NM Care Corner, to learn more about their symptoms A Spanish MyChart version of results will be available to Spanish-speakers if they endorsed Spanish as their preferred language in MyChart.

Participants in the post-implementation will follow the same protocol for the participation raffle.

A description of this clinical trial is available at *http://www.ClinicalTrials.gov* as required by U.S. Law**.**

Evaluation of Implementation. As with our prior work, we will work with EDW (a comprehensive and integrated repository of all NM clinical and research data sources that facilitates research, clinical quality, health care operations, and medical education) programmers to conduct queries of EHR data to produce reports of number of assessments initiated and completed, number of clinical alerts generated, and time to clinical response to generated alerts. Our evaluation plan is guided by Proctor et al.'s taxonomy of implementation outcomes, which includes acceptability, adoption, appropriateness, feasibility, fidelity, penetration, and sustainability. We will evaluate those implementation outcomes specific to the spread of NU IMPACT across the eight clinics in the NMHC. Consistent with the recommendations of Curran et al. and Santana and Feeny's model for evaluating ePRO clinical applications, we will conduct a process evaluation of implementation to inform the spread of NU IMPACT outside of Northwestern Medicine. We will use our conceptual framework for evaluating facilitators and barriers, the implementation plan, and patient- and system-level outcomes. As noted, implementation constructs often overlap and certain data contribute to one or more markers. EHR data can be used to determine adoption (use/intent to use) and penetration (proportion of patients reached) of the program in each clinic. These metrics directly relate to fidelity of implementation (universal screening; follow up for triggers approaching 100% saturation). Adoption is important at both the patient- (completing the screener) and provider-level (following-up with patients that trigger alerts). Similarly, penetration is determined by computing proportions at two levels: (1) patients enrolled and completing assessments from among the total number of patients approached to be part of the program; and (2) patients who are referred for appropriate services from among those that trigger an alert. We will calculate this second level of penetration using a 3- month sampling period, which more closely approximates the number of patients that require follow-up services at any given point. A limited data set containing EDW data on NMPRO questionnaire-eligible patients will also be evaluated to report descriptors of the implementation cohort. A data use agreement established between Northwestern Medicine and Northwestern University was amended in July 2023 to facilitate the transfer of EDW data for this study's non-consented patient cohort in a way that complied with Illinois state privacy law. This amended data use agreement outlined safeguards and appropriate use of such data, including zip codes and sensitive protected health information. In August 2024, the Northwestern Medicine Privacy Steward advised the study team that a recent amendment to Illinois state privacy law has negated the need for the 2023 data use agreement, and that a HIPAA Waiver of Authorization will now cover the release of protected information, most significantly zip code and sensitive protected health information. Per IRB guidance, the study team has requested waivers of consent and HIPAA authorization for this study cohort.

We will evaluate the acceptability, appropriateness, and perceived sustainability of the program with at least 100 clinical stakeholders (physicians, surgeons, advanced practice providers such as nurse practitioners or physician assistants, nurses/nurse navigators, hospital leadership, and

administrators) working across study sites by administering a brief outcome survey approximately 12 months after the launch of implementation for each clinical unit. Surveys will be administered electronically (via REDCap) to any person who is identified as a "cPRO" user by clinical site or administrative lead during the study implementation period.

Eligible participant contact information (name and NM email addresses as provided by the clinical leads at each site) will be shared with study staff prior to the 12-month mark of implementation. Staff will add participants to the REDCap enrollment log at the 12-month mark to cue distribution of the outcomes survey. Since we are requesting a waiver of signature and documentation of consent, the clinical stakeholder participants will indicate consent online by agreeing or disagreeing to the consent language presented in REDCap before the survey. If stakeholders provide informed consent, they will be auto-advanced to the survey. Study investigators have operationalized a 4-week window for study survey completion, such as that participants will have four weeks to complete the survey before the enrollment window "expires" and no further automated reminders will be issued through REDCap. Participants may also receive verbal and email reminders from the study team and clinical practice managers throughout the enrollment period. Participants will receive up to four reminders to complete the outcomes survey. If after four weeks the survey is still incomplete, the assessment may be left open at the discretion of the investigators.

Surveys will take approximately 20 minutes to complete, and participants will be compensated $50 via electronic gift cards for a survey submission.

The battery of measures to be completed by stakeholders will consist of:

a) Screening and General Introductory Questions (7 items)
   - These items, screening (2) and introductory (5), will assess a participant's clinical involvement with cPRO.

b) Sociodemographic Questions (10 items)
   - These items cover a demographic form meant to capture participant background information.

c) "Appropriateness" subscale, Organizational Change Recipients' Beliefs Scale (8 items)
   - The subscale of the Organizational Change Recipients' Beliefs Scale[1] reports internal consistency reliability (e.g., α = .89-.95 reported across several studies[1]) and includes item indicators such as "*the change we implemented was correct for our organization*."

d) "Supportive Leadership" subscale, Implementation Leadership Scale (12 items)
   - These items concern the degree to which a leader exhibits specific supportive behaviors[2]. The ILS has excellent reported internal consistency reliability (e.g., α = .95[2]) and includes item indicators such as "*supports employee efforts to use evidence-based practice*."

e) The Normalization Measure Development (NoMAD) questionnaire (23 items)
   - This measure is designed to assess adoption and implementation of a new healthcare element[3]. Respondents react to statements (e.g., "I can see the potential value of the dashboard to increase shared decision making for my clinical care") on a 7-point Likert scale (1 = "Strongly Agree" to 7 = "Never relevant at this stage").

f) The Clinical Sustainability Assessment Tool (CSAT) (21 items)

- This measure is designed to examine organization's clinical sustainability capacity[4]. Respondents react to statements (e.g., "The practice has evidence of beneficial outcomes") on a 7-point Likert scale (1 = "To little or no extent" to 7 = "To a very great extent").

<u>Supplement Aim 2:</u>

Since cPRO is already embedded in Epic and results are viewed by our oncology providers, the 4Rs decisional support system will have the following functionalities. First, when a patient first reports a PROMIS-PF score below 45 (indicative of moderate or severe impairment), cPRO will alert the oncology provider thus identifying the right patient at the right time. This will be done for all patients who complete cPRO, therefore this decisional support system will be will include consented and non-consented patients across NM health system. Second, the cPRO alert will trigger a Best Practice Alert (BPA) within Epic to present the provider with a set of triage questions that will guide the provider in asking the right questions to determine the right rehabilitation service (physical therapy, occupational therapy, speech therapy, and/or physical medicine and rehabilitation physician). Once the right rehabilitation service is selected, an order set will pop up for cancer rehabilitation referral. Some aspects will be auto populated (e.g., name, address, medical diagnosis) whereas other aspects will have drop-down menus to select discrete required elements of the referral (e.g., rehabilitation diagnosis, specific impairment, etc.). Once the order is completed, it will be forwarded to the NM Outpatient Rehabilitation Services Centralized Scheduling team to match the patient with the right clinic location close to the patient's home. To illustrate, for a provider with a patient with head and neck cancer who has difficulty swallowing due to cancer-related lymph\edema, the BPA will suggest referral to a speech therapist. The BPA will include the ICD-10 diagnoses of their cancer type and lymphedema. The NM Rehabilitation Services Centralized Scheduling will match the patient with a speech therapist who is also a certified lymphedema therapist in an NM clinic Outpatient Rehabilitation Services closest to her/his home. This work flow has been developed with NM IS and approved by the HSCCs. See figure X below.

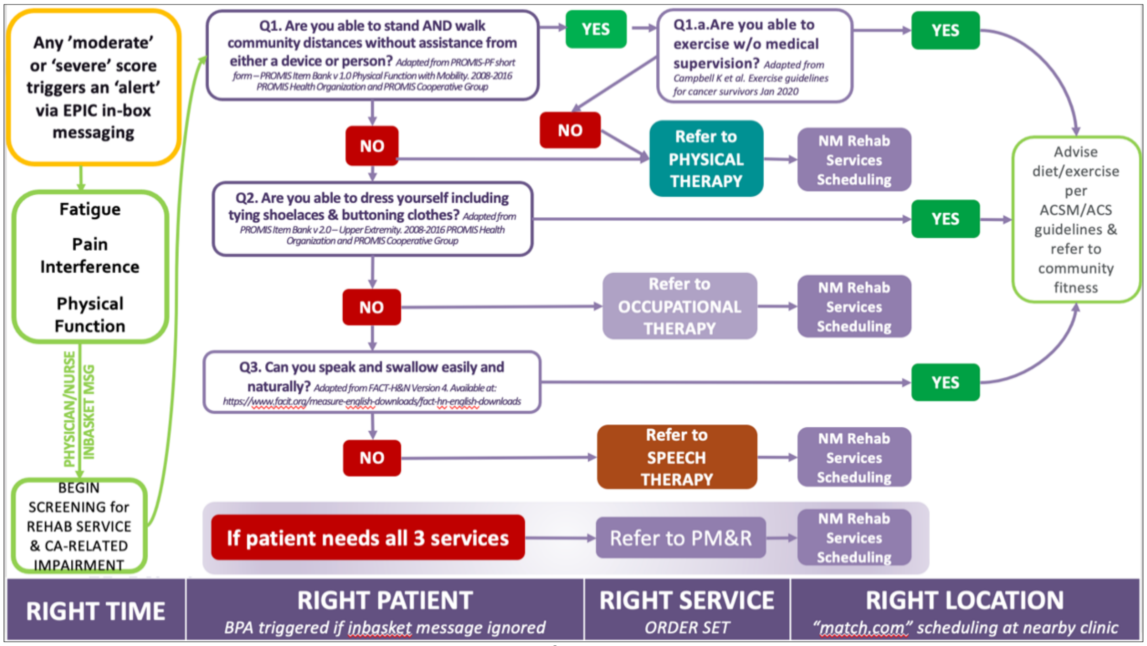

Figure X: 4Rs Cancer Rehabilitation Referral System - Clinic Workflow

Prior to the implementation of the 4Rs BPA across the system, we will conduct a series of (no more than 3) 2-4 week pilots of 1-2 types of clinicians from each NM region (Central, North, West and South). The clinicians will include oncologists, nurses and/or possibly physical therapists, community health workers, social workers, patient service representatives, advanced practice practitioners (APPs – nurses and physician assistants). The participating clinicians will receiving training on the BPA workflow, a copy of the 4Rs BPA Tip Sheet, and video on its use from the study team to implement it into their practice. The study team and NM IS will monitor the electronic workflow during the pilot time periods. This information will be supplemented with EDW data on the following BPA metrics:

1. #PROMIS-PF alerts & associated score
2. #PROMIS-PF alerts triggering BPA
3. Responsiveness to alert & BPA (dismissed v. response)
4. # alerts needed before BPA used for referral
5. Frequency & type of rehab service referred through BPA (PT, OT, ST, and/or PM&R)
6. # internal rehab referrals
7. # external rehab referrals
8. # of scheduled rehab visits resulting from rehab referral

Update on clinical procedures in the North region: The North region has opted to use manual entry of patients answers to cPRO with the help of a laminate that allows medical assistants to ask cPRO questions during clinic visits rooming. This laminate is approved for this work under the cPRO quality improvement initiative.

However, in order to pilot test the 4Rs system in the region, we are submitting the same laminate and manual entry for answers to the 4Rs triaging questions. Please note that we are using the same method already being used in the North region but are simply adding the 4Rs triaging questions to the current method.

<u>NU-IMPACT Aim 3</u>

In Aim 3 of the NU-IMPACT Study, we will identify facilitators and barriers to system-wide implementation and disseminate to other health systems. We will conduct stakeholder focus groups and patient-facing individual interviews to gather feedback from clinicians, administrators, and patients about cPRO implementation facilitators and barriers as well as experiences related to the study intervention (including in Spanish; ns =30, 10, & 50, respectively). Qualitative work will commence toward the end of study recruitment. We aim to recruit clinicians and patients cross-regionally to ensure representation from each NMHC clinic. All focus groups and individual interviews will be conducted via the online web conferencing and secure web-based platform Zoom. Focus groups and individual interviews will be audio recorded and transcribed after the sessions are completed. All meetings will be kept confidential by limiting attendees to study staff and eligible participants and password protecting all meeting links. Patients will only use their first

name or alias. Audio recordings will be saved in a password-protected file on the NU shared drive and will not be saved to the NU Zoom platform or "cloud".

Clinician-facing Focus Groups:
We will use a purposeful sampling strategy to recruit key stakeholders (clinicians and administrators) cross-regionally. Eligible stakeholders will be sent an email by the study PI to ask about potential interest in participation. Stakeholders may receive a reminder email if not response is received after 2 days (*see Clinical Stakeholder FG Communications – Study Invitation Email and Reminder Invitation Email*). All recipients of the recruitment email will be blind carbon copied (BCC'd) and focus group recruitment invitations will be sent out in batches of 6-12 people or one focus group at a time. Interested stakeholders will respond to the email at which point a study member will reach out to assess possible availability to join a focus group (*see Clinical Stakeholder FG Communications – Study Response Email*). Once a common availability is confirmed for all interested stakeholders a meeting time will be scheduled and research staff will collect informed consent (*see Clinical Stakeholder FG Communications – Consent Email*). Since we are requesting a waiver of signature and documentation of consent, the clinical stakeholder participants will receive an e-mail invitation with a link to the consent form. They can indicate consent online by agreeing or disagreeing to the consent language presented in REDCap. If stakeholders provide informed consent, they will be auto-advanced to a brief questionnaire to collect relevant sociodemographic and clinical experience information. Reminder emails will be circulated for potential participants that have not consented leading up to the scheduled date and time. A final Zoom conference link will be circulated once consent and demographics are obtained from stakeholders (*see Clinical Stakeholder FG Communications – FG Confirmation Email*).

We anticipate that each group will include between 6 and 12 participants and last between 60 and 90 minutes in duration. Team members with expertise in qualitative research (Allison Carol, Sofia Garcia, Betina Yanez) will conduct the focus groups, following a semi-structured interview guide. Clinicians and administrators will be compensated $100 for their time by electronic gift card. Data will be coded and analyzed by the research team using a directed content analysis approach that involves use of existing implementation research theory, prior results, and empirical frameworks to develop a coding scheme and hypothesize the relationship between codes. A de-identified transcript of one clinician-facing focus group will be shared with study consortium investigators for additional analysis using a structured codebook.

Patient-facing Interviews:
We will conduct patient-facing qualitative interviews across three cohorts. Participants in the first (cohort 1, enhanced-care user group) and second (cohort 2, enhanced care non-user group) cohorts will be selected from those who participated, and were randomized, to the enhanced care arm of the parent study. Participants in the third cohort (cohort 3, non-consented) will be selected from amongst those Spanish-speaking NM patients who self-identify as Hispanic/Latino and who were invited to participate in the study, but did not consent.

Cohorts 1 and 2:
Bi-lingual researchers will screen for eligibility from the list of study participants who were randomized to the enhanced care arm of the parent trial and completed the study in the past three months. E-mail invitations will be sent (in English or Spanish depending on the person's preferred language) with a brief overview of the study and a protected REDcap link to e-consent. Interested participants will use the link to provide informed consent and complete a survey collecting select

self-reported demographic (e.g., ethnicity, marital status, age) and clinical characteristics (e.g., cancer type; time since diagnosis). Additional questions regarding acculturation and technical literacy are also included. The survey is expected to take approximately 15 minutes. Those who consent will also be asked to participate in a semi-structured interview via Zoom Video Communications, expected to last 40-60 minutes. A telephone interview is acceptable for circumstances in which a participant is not able to access or use Zoom. Research staff will work with consented participants to arrange a mutually agreeable time for the interview and the interview will also be conducted in the participants preferred language. A second team member will participate to assist with note taking and technical issues should they arise. Team members will be trained in qualitative methods to conduct the interviews using a custom-built, standardized semi-structured guide Interviews will be audio recorded and transcribed and stored in password protected files. Topics will prioritize perceived barriers and facilitators to use of the enhanced care MyNM Care Corner website and overall experiences. Patients will receive $50 for their time via an e-gift card.

PLEASE NOTE:THIS SUBMISSION INCLUDES A RECRUITMENT SCRIPT, CONSENT FORM, SURVEY QUESTIONS, AND PAYMENT INFORMATION STANDARD TO COHORTS 1 and 2 AND THE SEMI-STRUCTURED INTERVIEW GUDE FOR COHORT 1 IN ENGLISH AND SPANISH. WE WILL SUBMIT A SEPARATE INTERVIEW GUIDE FOR COHORT 2 IN A SUBSEQUENT MODIFICATION.

Cohort 3:
We plan to conduct interviews for cohorts 1 and 2 first and then use what we learned to inform our approach for the interviews in cohort 3. The procedures, consent form and qualitative interview guide for cohort 3 will therefore be submitted in a subsequent modification.

Data Analysis:
We will code interview data using a directed content analysis approach that involves use of existing implementation research theory, prior results, and empirical frameworks to develop a coding scheme and hypothesize the relationship between codes. A codebook will be developed and codes will be refined using a subsample of focus group data. Subsequent focus group data will be coded by a team of coders. Twenty percent of the groups will be double-coded to calculate reliability. Disagreements will be resolved via expert consensus. In addition to preparing a manual of operations, and documenting all steps, procedures, challenges and opportunities that will be available for dissemination at the end of Year 6, we will hold open webinars presenting the implementation program, study outcomes and implications for implementation at other systems.

<u>Supplement Aim 3</u>

For the NU IMPACT Diversity Supplement activities, we will conduct additional focus groups with clinical stakeholders and patients who are involved in our pilot studies to receive feedback on implementation planning, usability, and identify potential adaptations for implementation and, if possible, dissemination. Participants may overlap with the parent study's (NU IMPACT) evaluation of implementation procedures. However, the NU-IMPACT Diversity Supplement (Supplement) study focus groups are separated from the parent study focus groups because the parent study is currently at the end-stage of the implementation process whereas the supplement study is in the pre-implementation stage. Additionally, there are important differences in the objectives of both the studies. The Supplement focus groups will be centered around how the 4Rs cPRO/BPA alerts assist in triaging, assessment of cultural readiness for the alerts system, and stakeholder

perceptions. We intend to conduct one clinician focus group within each region (a total of four focus groups) and one patient focus group with patients from across regions. This means we will conduct a total of 5 focus groups (4 with deliverers of the 4Rs system – physicians, advanced practice providers, nurses, medical assistants from the NMHC North, South, West and Central Regions and 1 with patients from all 4 regions). The focus groups will last for a duration of 60-90 minutes. Participants will be compensated $50 for their time. While we will make every effort to schedule our focus groups at a time convenient for all participants, it is possible that patients in particular may have scheduling conflicts that prohibit participating in a focus group. If this is the case, we will instead schedule a one-on-one interview at a time most convenient for the patient. All focus groups and/or interviews will take place over Zoom that is passcode protected. Based on the feedback we receive from the stakeholders; we will determine whether to conduct more pilot studies of the modified 4Rs BPA system or proceed with system-wide implementation. Dr. Ann Marie Flores (Principal Investigator- NU IMPACT Supplement) who is experienced in qualitative research will conduct the focus groups with a semi-structured interview guide (See attached Provider and Patient Facing Focus Group Interview Guides). The data obtained from the focus groups will be analyzed using content analysis with the help of study staff after the focus groups. The data will only be shared with the members of the study staff.

Data will be coded and analyzed by the research team using a directed content analysis approach that involves use of existing implementation research theory, prior results, and empirical frameworks to develop a coding scheme and hypothesize the relationship between codes.

9.0 Incomplete Disclosure or Deception:

N/A.

10.0 Recruitment Methods:

Aim 2

Participants will be drawn from the pool of NM cancer patients across the four regions. Specifically, patients of a clinician who has agreed to have his or her patients contacted and meet the eligibility criteria as stated above.

Each night, an EDW pull will flag patients based on the eligibility algorithm) based on clinical unit randomized rollout. Each day, this list of patients will automatically populate into a REDCap project. Logic will be included to remove duplicates. An email will be sent to the newly populated patients informing them of the project (see Survey Email Templates under the Recruitment Materials section of this modification). Each patient will receive a customized link. Patients who are interested in participating can click on that link, which will take them to REDCap to complete the consent form and study measures. We may also reach out by phone.

Patients who consent and complete the baseline survey will be sent additional customized links to complete the survey monthly after baseline for one year. For these surveys, patients will receive a reminder email or text message every 3 days for up to 4 reminders over 14 days, and we may also reach out by phone. These reminders will only be sent if the participant has not started their survey.

To enhance representation of Latino/a English and Spanish-speaking participants in our post-implementation phase, we plan to diversify our recruitment strategy by including some targeted in-person recruitment in the clinics. In partnership with our clinical lead and co-investigator Dr. Sheetal Kircher (who will be able to facilitate EPIC access to NM schedules for research staff with Level 3 access), we plan to identify eligible Latino/a English and Spanish-speaking patients from the standardized EDW pull that flags patients based on the eligibility algorithm (described above) and then approach patients in the clinic before their next scheduled appointment. The research team has requested and received EDW exception approval to utilize EPIC access for recruitment purposes and these details are in compliant with that request. Research staff will work with clinic leads to determine optimal recruitment locations, but we anticipate that this will likely happen in the waiting room or in a treatment room. Participants will be approached and asked if they would like to hear more about a research opportunity using the IRB approved text that is in the recruitment e-mails. If a patient is interested in participating, the coordinator will manually enroll and e-consent the patient using the standard approved platform in REDCap with an approved, password protected study lap-top computer. The participant will then receive information via e-mail on their randomization status and how to start completing forms. Patients who decline participation will be documented in a separate password protected tracking sheet.

<u>Aim 3</u>

- *Clinician Focus Groups*
  - Clinicians and administrators will be recruited using a purposeful sampling strategy to recruit key stakeholders cross-regionally. Stakeholders who have been exposed to clinical cPRO procedures for a minimum of one year will be considered an eligible and recruited by email to assess potential interest in participating in a focus group. Stakeholders will have the option to decline participation. Focus group meeting coordination will be arranged around interested participant's schedules.

- *Patient-facing Interviews*
  Cohorts 1 and 2:
  - Researchers will screen for eligibility from the list of study participants who were randomized to the enhanced care arm of the parent trial and completed the study in the past three months. Other eligibility criteria (see Section 3.0 Inclusion and Exclusion Criteria for Aim 3) will be used to determine cohort designation and the language (English or Spanish) used in participant communication, the survey and the qualitative interview. Eligible participants will be recruited via e-mail invitations and/or telephone outreach in the participant's preferred language (English or Spanish).

  Cohort 3: Relevant details will be provided in a subsequent modification.

<u>Supplement Aim 3</u>

- *Clinician Facing Focus Groups:*
  - The clinical stakeholders will be recruited via email using a purposive sampling strategy. They will have the right to decline participation.
- *Patient Facing Focus Groups:*

- All eligible patient participants will be identified by EDW reports that include name and contact information (phone number and email address). Patients will be recruited via email invitations and/or telephone outreach.
  - Patients consented under the parent study will be recruited via an EDW report that also contain additional patient identifiers such as patient ID, associated physician, 4Rs responses, details about the type of referral the patient received through the 4Rs BPA (physical therapy (PT), occupational therapy (OT), Speech Therapy (ST), and/or Physical Medicine & Rehabilitation physician (PM&R)) and date of referral.
  - We are requesting a partial HIPAA of Authorization Waiver for patients not consented under the parent study (NU-IMPACT). Contact information will be pulled for recruitment will only be retained for those who agree to participate in the study. An Appendix has been submitted with additional details.

11.0 Consent Process:

Aim 2

All patients who are determined to be eligible and are interested in the study will be instructed to click the REDCap link included in the email message informing them of the study. The patient email message will be in both English and Spanish. The clinician email will only be in English. The link will take them to the study's electronic consent form, which is housed in REDCap and includes the required language to obtain HIPAA Authorization. They will have the option to choose their preferred language to access either the English consent or Spanish Consent. The consent form also includes the contact information of study staff and the IRB. Participants will have the opportunity to ask any questions they may have before providing a written electronic signature at the end of the consent form.

The study team will also obtain a limited data set containing EDW data on members of a non-consented, implementation cohort. This cohort is comprised of NMPRO questionnaire-eligible patients who routinely receive the NMPRO cPRO questionnaire prior to oncology appointments as a standard of care. Their data will be evaluated to report descriptors of the implementation outcomes cohort.

We have requested waivers of consent and HIPAA authorization for the implementation outcomes patient cohort. This study presents minimal risk to patients. Northwestern University's Department of Medical Social Sciences has not encountered severe distress in the thousands of cancer patients and survivors who have completed self-report symptom questionnaires in previous studies. Of the more than hundreds of thousands of participants over 15 years, less than 1% have become emotionally upset to any extent. As risks are relatively low and are easily managed through referral to the cancer clinic's Supportive Oncology Program—and because benefits for participants are potentially substantial—the balance of risks-to-benefits are reasonable.

As in any research, there is a slight risk of loss of confidentiality. Measures taken in this study to protect patient privacy and confidentiality of self-report and other data are outlined in Section 16

of this protocol (Provisions to Protect the Privacy and Confidentiality of Participants and the Research Data). Moreover, the requested waivers of consent and HIPAA authorization will not adversely affect the rights and welfare of patients in the implementation outcomes patient cohort. Details of this study's data monitoring plan to ensure the safety of all study participants, including those in the implementation outcomes patient cohort, are outlined in Section 17 of this protocol.

Data obtained from the large (12,000+) implementation outcomes cohort is essential to evaluating whether the implementation strategies deployed through this study promote greater patient adoption of cPRO in standard of care practice and improve health outcomes. Data on this cohort will include zip code, which is required to facilitate health equity analyses. Aside from zip code, all identifiers will be removed by EDW programmers prior to sharing data with the analysis team and with the NCI IMPACT Consortium.

Aim 3

- *Clinician Focus Groups*
    - All focus group interview participants will be required to complete an online informed consent through REDCap prior to engaging in focus group activities. This online consent will be administered before participants are asked to complete the clinical stakeholder demographic survey. Participants will have the opportunity to ask questions before consenting to study procedures. Copies of consent forms will be provided to participants to review in advance and keep in their personal records. Consent forms will include contact information of study staff and the Northwestern University IRB.
- *Patient-facing Interviews*
    - Interview participants across all three cohorts will be required to complete an online informed consent through REDCap prior to engaging in any research activities. Participants will have the opportunity to ask questions before consenting to study procedures in their preferred language (English or Spanish). A copy of the consent form in their preferred language will be provided to participants to review in advance and keep in their personal records. Consent forms will include contact information of study staff and the Northwestern University IRB.

Supplement Aim 3

All eligible and willing participants will be required to complete an informed consent form administered electronically via REDCap prior to their participation in the focus groups. They will have the opportunity to ask questions about the focus groups at the time of consenting. They will also have a copy of their consent forms for their own records. Consent forms will include contact information of study staff and the Northwestern University IRB.

12.0 Financial Compensation:

Aim 2

Participants completing study assessments will be eligible for a quarterly raffle for all NM regions. For each REDCap assessment completed, a participant will be enrolled in a raffle for a $100 Amazon gift card. That is, if a participant completes the baseline assessment and the 3-month assessment, then the participant's name will be included in the raffle pool twice. During the participant enrollment phase for Aim 2, the participation raffle will be held quarterly (from start date of participant enrollment) for all study regions until the end of the projects participant data collection phase. Only prospective assessments during the quarter will be eligible to be included in that quarter's participant raffle. There will be five winners per quarter. Information on how to access the Amazon gift card code will be emailed to participants within approximately one week of the raffle. Within the week of the email notice being sent, the study team will call participant winners by phone and to confirm receipt of the Amazon gift card.

Clinical stakeholder participants completing REDCap outcomes assessments will be compensated $25 for their participation in the study. Clinical participants will either receive compensation via electronic gift cards. Information on how to access electronic gift card funds will be provided to participants at time of compensation.

Aim 3

- *Clinician Focus Groups*
    - Clinical and administrative participants who attend a focus group interview will be compensated $100 for their time and participation. They will receive compensation via electronic gift cards. Information on how to access electronic gift card funds will be provided to participants at the time of compensation.
- *Patient-facing Interviews*
    - Participants across all three cohorts who consent to the study and complete the interview will be compensated $50 for their time and participation. They will receive compensation via electronic gift cards. Information on how to access electronic gift card funds will be provided to participants at the time of compensation, in their preferred language (English or Spanish).

Supplement Aim 3

Participants will receive compensation in the form of a $50 gift card. They will receive the gift cards electronically and will be provided with information on how to access their gift cards.

13.0 Audio/Video Recording

Aim 3

- *Clinician Focus Groups*
    - Focus groups will be audio recorded using the web-based video conferencing platform Zoom, for the purpose of retaining information not captured in notes collected by study staff, transcription, and for later data analysis. Only project

staff will have access to recordings which are to be stored in secure computer drives and Northwestern University's secure servers. All data will de-identified after interviews are completed and audio recordings are transcribed. Original recording files will be deleted after being transcribed and saved in secure locale. Recordings will not be shared or distributed for purposes other than data analysis.

- *Patient-facing Interviews*
  - Individual interviews across all three cohorts will be audio recorded using the web-based video conferencing platform Zoom Video Communications, for the purpose of retaining information not captured in notes collected by study staff, transcription, and for later data analysis. If a participant is limited to telephone participation, a recording device will be used for the audio recording. Only project staff will have access to recordings which are to be stored in secure computer drives and Northwestern University's secure servers. All data will be de-identified after interviews are completed and audio recordings are transcribed. Original recording files will be deleted after transcription and saved in secure locale. Recordings will not be shared or distributed for purposes other than data analysis. Audio recordings in Spanish will be translated to English to facilitate coding.

<u>Supplement Aim 3</u>

Focus groups will be conducted via Zoom and recorded with the Zoom audio feature. Focus groups will be audio recorded and transcribed after the sessions are completed. Audio recordings will be deleted after being transcribed. All meetings will be kept confidential by limiting attendees to study staff and eligible participants and password protecting all meeting links. Participants will only use their first name or alias. Transcriptions will only use a series of the first letter of the name in order to distinguish the comments made by each individual to ensure that we capture information on distinct individual experiences. For example, a participant whose first names starts with the letter "A" will be identified as "AAAA" in the transcript. If we have more than 1 participant with the same first letter of the name used, we will identify them as "AAAA1" and "AAAA2" and so forth. Focus group recordings will not be saved to the NU Zoom platform or "cloud".

14.0 Potential Benefits to Participants:

Participants may not personally benefit from involvement in this study, but previous experience predicts that some participants will find reporting their symptoms and discussing their health care experiences to be helpful and satisfying. Reporting symptoms may improve the speed and extent to which they are triaged and responded to by health care providers.

15.0 Risks to Participants:

This project presents minimal risk to participants in receiving the enhanced care (EC) intervention, completing self-report measures or participating in focus groups (or individual interviews). In the over 3,000 patients assessed through our prior pilot work, there have been no reported incidents of distress or other adverse events. Additionally, Northwestern University's Department of Medical Social Sciences has not encountered severe distress in the thousands of cancer patients and survivors who have completed self-report symptom assessments or focus groups / qualitative

interviews in previous studies. Of the more than hundreds of thousands of participants over 15 years, less than 1% have become emotionally upset to any extent. Lastly, as in any research, there is a slight risk of loss of confidentiality. Measures taken in these rare instances are described below. Participants randomized to the EC condition will receive information (e.g., tools, modules, skills) specific to improving self-management skills, gaining knowledge regarding their medical condition, improving communication skills and empowering them to improve motivation and self-management. In rare cases, a participant may feel overwhelmed by such information and experience transitory distress states. However, this has not been our experience in multiple web-based studies that the study team has conducted. Nonetheless, regardless of study cohort or intervention arm, participants are instructed to call study staff with any concerns or questions they may have.

16.0 Provisions to Protect the Privacy and Confidentiality of Participants and the Research Data:

All sources of information will be coded using a special participant number, which precludes it being matched with identifying information. Issues surrounding confidentiality are of utmost importance. In all cases, highly personal and clinical information will be obtained. The participants will sign a statement attesting to their understanding that the information they provide will be held as personal and confidential to the extent permitted by law. Access to the computer data files will be by password codes. Participant consent and data will be collected and stored on e-consent, a research management tool developed in the PI's home department, which enables secure study-specific websites. All data will be stored on password protected, secure servers at Northwestern University. Data management strategies will take full consideration of regulations on the security and confidentiality of electronic data.

Summary data reports will not contain any identifying information. Upon completion of the study and data retention period, patient identifiers will be deleted in compliance with the Health Insurance Portability and Accountability Act. Though we regard the risks of participating in this study as very low, we will take care that every recruiter, project personnel, etc. is trained to handle situations sensitively and with empathy. All research staff are required to complete NU mandated training on the Protection of Human Research Participants, and this training experience must be renewed on a yearly basis. Certificates for all research staff will be kept on file in the PI's office at each respective site. As risks are relatively low and easily managed, and because benefits for participants are potentially substantial, the balance of risks-to-benefits are reasonable. Despite extremely low incidence in related prior studies, it is conceivable that a participant may later become severely depressed or otherwise distressed while on study. Therefore, as we have in prior studies, participants who report significant distress (in their assessment measures or communications with study staff) will be referred to the cancer clinics' Supportive Oncology Program. Drs. Garcia, Yanez, and Penedo (both clinical psychologists) will ensure that study staff are trained to monitor for significant patient emotional distress or physical symptoms that merit referral (and how to make those referrals). Study staff will be instructed on the appropriate courses of referral if a participant is considered to be at risk for a safety concern (using the existing Supportive Oncology Crisis Protocol available at each clinic). Telephone and pager numbers for members of the Supportive Oncology Team as well as how to contact on-call oncology providers will be kept on file to be used in the event of any mental or physical health emergencies. Our study

team (Cella, Yanez, Garcia, Penedo) also has crisis intervention training and, if warranted, can assist with clinical assessment (e.g., suicidal ideation, plan of action, and likelihood of carrying out the plan).

Patient self-report data, participant tracking and flow, and EHR data will reside in our Electronic Data Warehouse and Northwestern University Clinical and Translational Sciences (NUCATS) Institute's REDCap instance, which are securely stored at the Northwestern University Research Data Center. The data management environment meets the security requirements identified in the Agency Automated Information Systems Security Program (AISSP) Handbook. REDCap requires use of current passwords and log-on codes to protect sensitive AIS (Automated Information System) data from unauthorized access. All system access requires a user name and password. All users who require anything more than "internet user" security to the Northwestern University campus network must have a unique ID assigned. The Northwestern ID is used to control access to data files and applications that reside on the network. Every Northwestern ID is required to have a complex password assigned. Restriction to REDCap involves maintaining an encrypted list of current users and authorizations based on permissions and roles assigned to each user. Northwestern University provides REDCap administrators with secure Virtual Private Network (VPN) access. Operating system and database permissions are set by the REDCap administrators. User accounts have limited system resource access. The availability and flow of data is limited to the security access identified and signed off by management, and is controlled by the database administrator who pre-defines security access levels. This ensures that only data necessary for that individual's work function are viewable. All requests are validated before attempting to submit data to the database. Any internal or stored reference to a participant is accomplished through a unique identifier key. Data are stored and accessed in full compliance with institutional IRB and governmental regulations regarding privacy and security. Northwestern University has a secure data center that houses all servers. This center is limited to authorized employees via the use of a card swiping system and biometric reader on the entrance doors. Northwestern University's Department of Medical Social Sciences has established standard operating procedures relevant to data retention and removal based on NIH data retention requirements. Sensitive participant identifiers of on-going studies are encrypted and kept confidential. This procedure was initially designed to comply with HIPAA requirements. Terminated studies have all participant identifiers removed from electronic media storage after the retention period. Hard copy documents with participant identifiers will be shredded in a timely manner. Northwestern University provides comprehensive data backup. All data is stored in a dedicated storage server on top of redundant disk array. A snapshot of the virtual machines is taken every night and stored on backup storage that is alternated every two weeks. A full backup of the database is taken every night with incremental backups taken every two hours. Communications lines terminate in locked PBX rooms and in the data center. Contingency plan for disaster recovery is in place. The Northwestern University Research Data Center has two Uninterruptible Power Supplies and a Diesel generator in the event of a power shutdown. These units are tested on a routine basis.

17.0 Data Monitoring Plan to Ensure the Safety of Participants:

The Data Safety and Monitoring board (DSMB) will be chaired by Masha Kocherginsky, PhD, Professor (Biostatistics), Department of Preventive Medicine, and Professor (Gynecologic Oncology), Department of Obstetrics and Gynecology, Feinberg School of Medicine, an

experienced cancer biostatistician with the primary research focus in the design and analysis of clinical trials. Dr. Kocherginsky is also Director of the Quantitative Data Sciences Core at the Robert H. Lurie Comprehensive Cancer Center at Northwestern University, a shared resource that provides support to cancer researchers in the areas of biostatistics, bioinformatics and clinical informatics. The DSMB will also include Ruth Carlos, MD, Professor (Radiology), Division of Abdominal Radiology, Michigan Medicine. Dr. Carlos is the Assistant Chair for Clinical Research at the University of Michigan and has expertise in multi-disciplinary, multi-center clinical trials on prevention, surveillance and cancer care delivery. The final member is Lynne Wagner, PhD, Professor (Health Policy and Management), UNC-Chapel Hill. Dr. Wagner is a licensed clinical health psychologist with expertise in psychosocial oncology and measuring patient-centered outcomes in oncology. None of these DSMB members are associated with the proposed work and all have DSMB experience. The DSMB will meet semi-annually via WebEx, beginning in the fourth quarter of year 1. Occurrence of unexpected serious adverse events (e.g., hospitalization, injury, or death of a study participant) will be discussed at weekly project meetings. Adverse events will be documented, logged and (those meeting the criteria of 'Reportable New Information") submitted to the NU-IRB in writing as mandated. The PI will review data safety quarterly, and a cumulative report will be submitted an annual basis as required for continuing review and/or final report. Interim study analyses for the purpose of discontinuing the randomized arm of the study will only be conducted if during a review of adverse events it seems that risks or complications appear to be greater than those originally stated. If the PI suspects that an unusual number of adverse events is occurring, our Statistician, Dr. Scholtens, in consultation with Dr. Kocherginsky, will break the condition assignment and participant identifier code and determine if the adverse events occur significantly more frequently in any one condition. The DSMB will be convened at this point (in addition to biannual meetings) to address unexpected complications. As the potential source of adverse events is likely to come from psychosocial assessment, or the self-management intervention, our statistician will attempt to assess any relationship between these factors and adverse events. In the unlikely event of such analyses, the IRB will be kept informed of the results in a timely manner. Moreover, the IRB will be notified within one working day of any temporary or permanent suspension of the study protocol. Adherence to the protocol as well as data accuracy and security are detailed in the protocol and informed consent document. No study personnel or members of the DSMB have a conflict of interest with the study protocol.

18.0 Data, and if applicable, Specimen Banking:

N/A.

19.0 Data Sharing:

The IMPACT Consortium consist of three Research Centers and a Coordinating Center, which works collaboratively to conduct evidence-based research to improve symptom control for cancer patients and engaged in developing common data elements across Research Center for further analysis. A requirement of this cooperative NCI Moonshot grant is multi-level data sharing. This will include sharing limited datasets for consortium-level analysis, as well as de-identified datasets for restricted and unrestricted public use. All shared data will be maintained using secure, password-protected repositories. All published data will be reported in aggregate.

Limited dataset on consented patients:

The research team will share a limited dataset with the IMPACT Consortium. With the exception of zip code which is needed for consortium level health equity analysis, we will remove or code any direct identifiers (such as name, dates, address, telephone or social security number) before files are shared with other researchers to ensure that, by current scientific standards and known methods, no one will be able to identify these participants from the information we share. See Table 3 below for list of variables.

Table 3: Limited Dataset shared with Consortium on Consented Patients (patients who consented during pre-implementation and post-implementation)

Content Area	Description	Field examples
Demographics	Demographics of study population, one row per patient	site, age (in days) at the time of consent, sex, race, ethnicity, marital status, education, employment, insurance, language, interpreter needed, 5-digit zip code
MyChart	Info on MyChart enrollment, multiple rows per patient	Days from consent enrolled in MyChart, days from consent accessed, days from consent discharge, Mode of Access
Encounter	Encounters documented in EHR, multiple rows per patient	encounter dates, encounter type, appointment date, department ID, provider ID, discharge date
Cancer Diagnoses	Information on cancer diagnoses, multiple rows per patient	Encounter ID, provider ID, stage of cancer, system of staging, grade of cancer, grading system, indicator for first diagnosis
Diagnoses	All diagnoses on problem list/encounter diagnoses	Encounter ID, diagnosis date, provider ID, diagnosis code, diagnosis code system,
Procedures	All procedures in EHR	Encounter ID, procedure date, provider ID, procedure code, procedure code system,
Cancer Medication	All cancer meds from beacon treatment plan	Medication code, start/end date, dose, route of administration, treatment intent
Symptomatic Medication	All medication listed in EHR	Medication code, start/end date, dose, route of administration
Vital Signs	BMI, multiple rows per patient	BMI, measure date
PROMIS measures	All cPRO completions	cPRO date completed, scores for 5 domains
REDCap study data	All surveys in REDCap	
Study specific fields	Indicators for if Pre/Post I, randomized arm, site, cancer continuum, one row per participant	
SDoH	zipcode, one row per participant	zipcode 5 digit
NPI	Info from NPI - can merge on provider ID to get info on providers	

Limited Dataset on Non-Consented Patients:

In addition, a limited dataset on all NMPRO-eligible patients of study implementation will be extracted from the EHR and shared with the NCI IMPACT Consortium. As stated earlier, a data use agreement established between Northwestern Medicine and Northwestern University was amended in July 2023 to facilitate the transfer of EDW data for this study's non-consented patient cohort in a way that complied with Illinois state privacy law. This amended data use agreement outlined safeguards and appropriate use of such data, including zip codes and sensitive protected health information. In August 2024, the Northwestern Medicine Privacy Steward advised the study team that a recent amendment to Illinois state privacy law has negated the need for the 2023 data use agreement, and that a HIPAA Waiver of Authorization will now cover the release of protected information, most significantly zip code and sensitive protected health information. Per IRB guidance, the study team has requested waivers of consent and HIPAA authorization for this study cohort. A data use agreement established between Northwestern University and the NCI IMPACT Consortium at the outset of the study serves as the pathway for Northwestern University to share this limited data set with the NCI IMPACT Consortium.

Aside from zip code, all identifiers will be removed by EDW programmers prior to sharing data with the analysis team and with the NCI IMPACT Consortium. See Table 4 below for list of variables.

Table 4. Limited dataset to be Shared with Consortium on Non-consented Patients* (Implementation populations during pre-implementation and post-implementation)

Content Area	Description	Field examples
Demographics	Demographics of study population, one row per patient	site, age in days from index, sex, race, ethnicity, marital status, education, employment, insurance, language, interpreter needed, 5-digit zip code
MyChart	Info on MyChart enrollment, multiple rows per patient	Days from index enrolled in MyChart, days from index accessed, days from consent discharge, Mode of Access
Encounter	Encounters documented in EHR, multiple rows per patient	encounter days from index, encounter type, appointment date, department ID, provider ID, discharge days from index
Cancer Diagnoses	Information on cancer diagnoses, multiple rows per patient	Encounter ID, provider ID, stage of cancer, system of staging, grade of cancer, grading system, indicator for first diagnosis
Diagnoses	All diagnoses on problem list/encounter diagnoses	Encounter ID, diagnosis days from index, provider ID, diagnosis code, diagnosis code system,
Procedures	All procedures in EHR	Encounter ID, procedure days from index, provider ID, procedure code, procedure code system,
Cancer Medication	All cancer meds from beacon treatment plan	Medication code, start/end days from index, dose, route of administration, treatment intent
Symptomatic Medication	All medication listed in EHR	Medication code, start/end days from index, dose, route of administration
Vital Signs	BMI, multiple rows per patient	BMI, measure days from index
PROMIS measures	All cPRO completions	cPRO completed (days from index), scores for 5 domains

Study specific fields	Indicators for if Pre/Post I, randomized arm, site, cancer continuum, one row per participant	
SDoH	zipcode, one row per participant	zipcode 5 digit
NPI	Info from NPI - can merge on provider ID to get info on providers	

Underlying Primary Data for NU-IMPACT Publications:
To comply with an NCI requirement for all Cancer Moonshot-funded studies, NU IMPACT and the other IMPACT Consortium research centers will deposit de-identified analysis datasets used to create study publications in a data repository of the study team's choosing upon manuscript publication. The NU IMPACT study team will utilize Microsoft Dataverse for this purpose.

Public Use Dataset(s):
Lastly, the research team will select variables to be shared with the IMPACT Consortium for public access. This effort will meet the Consortium's request for research sites to provide a dataset at the end of the study for 'Restricted Public Use' and a second dataset for 'Unrestricted Public Use.' Details on specific variables will be updated in the protocol when confirmed by NCI and the IMPACT Consortium.

NU IMPACT data will be shared with the IMPACT consortium through the IMS IMPACT portal where files will be uploaded. A password-protected zip file will be created that contains the data and documentation file(s) using AES encryption using 7-Zip. The file will be uploaded to the IMS IMPACT portal by the NU IMPACT statistical analyst. The statistical analyst will call the IMS Senior Systems Analyst who manages the portal uploads with the password information so that they can download/open the files. A Data Use agreement will be executed to facilitate sharing this data wth the IMPACT Coordinating Center

During this study, participants may visit a Northwestern Memorial Healthcare Corporation entity (for example, Northwestern Memorial Hospital, Prentice Women's Hospital, Delnor Hospital, and Lake Forest Hospital) for research appointments. When this is occurs, these services may be scheduled or notated in the NMHC computer system. When a clinical exam or lab is done by NMHC or one of its employees for the purpose of this research study, that information will be kept in both NMHC's clinical records and in the study records.

Health information obtained for the purpose of this research study may be shared with the following offices or entities outside of Northwestern University and its clinical partners (or affiliates): the Northwestern University Institutional Review Board Office and Office for Research Integrity; the US Office of Research Integrity; the US Office for Human Research Protections; the US Food and Drug Administration.

Any research information shared with outside entities will not contain participant names, addresses, telephone or social security numbers, or any other personal identifier unless disclosure of the identifier is necessary for review by such parties or is required by law or University policy (except that such information may be viewed by the Study sponsor and its partners or contractors at the Principal Investigator's office).

20.0 Qualifications to Conduct Research and Resources Available:

The 18 key personnel named in this proposal are ideally suited to this project, covering all of the necessary disciplines. David Cella, PhD, PI, has had 30 years of continuous NIH funding, focusing on quality of life assessment, clinical trial outcome evaluation, and supportive care interventions, primarily in oncology. He will participate in the activities of all three units.

The Administrative Unit is led by Sofia Garcia, PhD, a licensed clinical psychologist who has pioneered various novel supportive care and survivorship interventions at NM. Dr. Garcia is experienced in managing complex multi-site projects that require coordination and communication across clinical, functional, and research units. She has a goal- oriented problem-solving style, and organizational skills that are well-suited to leading this unit.

The Design and Implementation Unit is led by Frank Penedo, PhD, a renowned clinical psychologist and clinical trial methodologist who specializes in promoting quality of life in African American and Hispanic cancer patients. Betina Yanez, PhD, is co-leader responsible for overseeing the intervention details. She is also a clinical psychologist who has studied psychosocial and treatment adherence challenges faced by women with breast cancer. Justin D. Smith, PhD, is also a co- leader and will be responsible for the implementation research aspects of the study. He is an experienced implementation scientist in NU's Center for Prevention Implementation Methodology. Karl Bilimoria, MD, MS, Professor and Vice Chair for Quality, Department of Surgery, is also Vice President for Quality at NMHC. He will be instrumental in providing leadership and engagement among faculty at the clinic level. Cynthia Barnard, PhD, is also Vice President for Quality, NMHC. She specializes in quality improvement methods within a patient-centered framework. She will provide critical input to the implementation strategy, intervention materials, and clinic issues.

The Data, Statistics and Informatics Unit is led by Denise M. Scholtens, PhD, a senior statistician expert in large, complex data analysis. The unit is co-led by Michael Bass, MS, an experienced informaticist and software developer. They have complementary skills ideal to effectively run this critical unit. Michael Kallen, PhD, MPH, is a psychometrician and statistical analyst who is highly qualified to plan and analyze the patient-reported and other outcome data. Firas Wehbe, MD, PhD, is Chief Research Informatics Officer at Feinberg School of Medicine. A skilled informatician, he will ensure optimal connectivity and compatibility of IT between the university and the health care system. Luke Rasmussen, MS, is a software engineer and database manager who has worked with Bass on creative HIT solutions for PROMIS and Infobutton implementations, will manage project data and help design connecting IT with solutions as needed.

Study Staff is led by Mary O'Connor, a clinical research associate with expertise is clinical research management and her team of clinical research staff.

There are six clinical colleagues (hematology/oncology, radiation oncology and gynecologic

oncology) who are key to the success of this project. Sheetal Kircher, MD, represents the Central region hematology/oncology clinical unit, and will serve on the NU IMPACT Executive Committee. With Dr. Kircher, we include five clinical investigators committed to providing quality care, including this integrated supportive care program. These collaborators are leaders at the clinical units we will randomly sequence into implementation: Dean Tsarwhas, MD, Barbara Buttin, MD, Christopher George, MD, and. They will advise on the clinical appropriateness of interventions and outcomes as they are operationally defined for the project roll-out, and they will also ensure support from the professional and clerical staff at the six respective clinics. In later years, they will participate in dissemination through meeting presentations and as coauthors on publications.

The **Diversity Supplement sub-study** is led by Ann Marie Flores, PT, MSPT, PhD, CLT. Dr. Flores is a medical sociologist, physical therapist, and certified lymphedema therapist who specializes in cancer rehabilitation research. She is an Associate Professor in the Department of Physical Therapy and Human Movement Sciences, Medical Social Sciences and is the Director of Cancer Rehabilitation for the Robert H. Lurie Comprehensive Cancer Center's Cancer Survivorship Institute.

References:

1. Armenakis, A. A., Bernerth, J. B., Pitts, J. P., & Walker, H. J. (2007). Organizational change recipients' beliefs scale: Development of an assessment instrument. *The Journal of applied behavioral science*, *43*(4), 481-505.
2. Aarons, G. A., Ehrhart, M. G., & Farahnak, L. R. (2014). The implementation leadership scale (ILS): development of a brief measure of unit level implementation leadership. *Implementation Science*, *9*(1), 1-10.
3. Finch, T. L., Girling, M., May, C. R., Mair, F. S., Murray, E., Treweek, S., ... & Rapley, T. (2018). Improving the normalization of complex interventions: part 2-validation of the NoMAD instrument for assessing implementation work based on normalization process theory (NPT). *BMC medical research methodology*, *18*(1), 1-13.
4. Malone, S., Prewitt, K., Hackett, R., Lin, J. C., McKay, V., Walsh-Bailey, C., & Luke, D. A. (2021). The Clinical Sustainability Assessment Tool: measuring organizational capacity to promote sustainability in healthcare. *Implementation science communications*, *2*(1), 1-12.

NU IMPACT Statistical Analysis Plan

1.0 INTRODUCTION

This Statistical Analysis Plan (SAP) for the Northwestern University IMPACT Research Center (NU IMPACT) study includes all plans for data summary and statistical analyses in support of NU IMPACT primary, secondary and exploratory hypotheses. A summary analysis plan and sample size justification are included in the NU IMPACT Protocol. The SAP is intended to provide a more detailed description of planned analyses. The SAP is not considered part of the protocol. Thus, modifications to the SAP do not require protocol amendments.

2.0 OBJECTIVES AND OUTCOME MEASURES

The overarching goal of the NU IMPACT study is to implement and evaluate a fully integrated oncology symptom assessment and management program across the Northwestern University (NU) affiliated healthcare delivery system, called Northwestern Medicine Health Care (NMHC). The legacy program, considered 'usual care' (UC), was developed under NCI grant CA60068 for medical oncology practice.[1-3] Based on its clinical appeal, it was adopted as a supportive oncology tool in gynecologic oncology.[2,4] Subsequent extension into hematology/oncology in our flagship hospital was then followed by full integration of the program into an expanded, single EHR (Epic) installation across all of NMHC. This system-wide installation, referred to as Northwestern Medicine Patient Reported Outcomes (NMPRO), went live in March 2018. The cancer center-specific version of the broader NMPRO system is referred to as 'cPRO'. The NU IMPACT study utilizes a hybrid type 2 effectiveness-implementation design with two complementary design features to address implementation and effectiveness research questions independently and simultaneously. This design will allow for evaluation of the impact of our implementation strategy on system-level outcomes, along with a sufficiently powered group-based comparison to demonstrate effectiveness on patient-level outcomes.

First, to best evaluate *implementation* outcomes, we will use a stepped-wedge trial design involving seven clusters and a total of 32 clinics (range: 1-9 clinics per cluster; see Figure 1). This will allow within- and between-site evaluation of system-level changes (e.g., patient cPRO adoption rates) due to prospective introduction of implementation strategies in an observational group of patients completing cPRO assessments (and being triaged and referred for relevant clinical services based on their assessment results) under UC in pre-implementation (pre-I) and post-implementation (post-I) phases. We will also identify patient- and system-level facilitators and barriers to implementation. Successful strategies and guidance on addressing barriers may be disseminated to other health systems.

Second, to demonstrate *effectiveness* of an enhanced care (EC) intervention for improving patient reported outcomes (PROs) compared to UC, our design also includes a smaller, embedded patient-randomized trial in which consented patients enrolled during post-implementation are randomly assigned to EC or UC and agree to complete a set of PRO surveys, with some administered monthly and others quarterly. The EC intervention includes additional self-management information through a patient-centered website and smartphone application called MyNM Care Corner on patient outcomes. During post-implementation, EC becomes available using a 1:1 randomization scheme (EC:UC) to new, consenting patients. Eligible patients who are identified during the pre-implementation phase are invited to consent to the same set of monthly and quarterly PRO surveys used during the patient-level randomized trial; those who do are not eligible for randomization during the post-implementation period.

The primary, secondary and exploratory objectives and outcomes for the NU IMPACT study are described below.

Primary Objectives and Outcome Measures

Primary objectives of the NU IMPACT study:

- To evaluate the effect of a system-level multicomponent package of implementation strategies for oncology symptom assessment and management at NMHC to increase adoption at the patient level
- To evaluate the effectiveness of EC compared to UC for improving PROs for cancer patients at NMHC

Primary implementation outcome in NU IMPACT:

- Patient-level adoption measured by the proportion of patients completing cPRO among those eligible for cPRO

Primary effectiveness outcomes in NU IMPACT:

- Individual-level PROMIS measures collected at baseline and monthly up until 12 months after enrollment
    - PROMIS Anxiety v1.0
    - PROMIS Depression v1.0
    - PROMIS Fatigue v1.0
    - PROMIS Pain Interference v1.1
    - PROMIS Physical Function v1.2

Secondary Objectives and Outcome Measures

Secondary objectives of the NU IMPACT study:

- To evaluate the effect of a system-level multicomponent package of implementation strategies for oncology symptom assessment and management at NMHC to increase reach in cPRO completion at the patient level
- To evaluate the effect of a system-level multicomponent package of implementation strategies for oncology symptom assessment and management at NMHC to increase cPRO adoption at the provider level
- To evaluate the effectiveness of EC compared to UC for improving healthcare utilization
- To evaluate the effectiveness of EC compared to UC for improving cancer treatment delivery
- To evaluate the effectiveness of EC compared to UC for improving cancer treatment satisfaction
- To evaluate the effectiveness of EC compared to UC for improving cancer patient self-management
- To evaluate the effectiveness of EC compared to UC for improving cancer patient toxicity
- To evaluate the effectiveness of EC compared to UC for improving cancer patient physical health

Secondary implementation outcomes in NU IMPACT:

- Patient-level reach measured by the proportion of patients enrolled in cPRO among those eligible for cPRO
- Patient-level reach measured by the proportion of patients who are referred for appropriate services from among those that trigger an alert in cPRO
- Provider-level adoption measured by the proportion of unique clinicians who use a cPRO dot phrase to follow up on triggers received from patients
- Survey outcomes evaluating appropriateness, acceptability, feasibility, and sustainability of the implementation strategies package supporting cPRO
- Qualitative data from focus group interviews concerning barriers/facilitators, acceptability, feasibility, and sustainability

Secondary effectiveness outcomes in NU IMPACT:

- Healthcare utilization metrics ascertained via the electronic medical record for within-NMHC utilization and by data collection directly from patients for outside-NMHC utilization, collected at 6 and 12 months
    - Number of emergency room/urgent care visits
    - Number of hospital admissions
    - Hospital days of stay
    - Number of unscheduled visits
    - Number of supportive care visits
- Cancer treatment delivery metrics ascertained via the electronic medical record for within-NMHC utilization within 12 months of enrollment
    - Number of treatment breaks
    - Number of changes in regimen
- Cancer treatment delivery satisfaction, collected via survey at baseline and quarterly
    - CAHPS
    - Collaborate
- Health related quality of life, collected via survey at baseline and quarterly
    - FACT-G7
- Symptoms and toxicities, collected via survey at baseline and quarterly
    - PRO CTCAE
- Physical activity, collected via survey at baseline and monthly
    - GODIN
- Financial burden, collected via survey at baseline and quarterly
    - COST Facit V2
- Self-management metrics, collected via survey at baseline and quarterly
    - PROMIS Self Efficacy Manage Meds/Tx SF 4a v1.0
    - PROMIS Self Efficacy Manage Symptoms SF 8a v1.0
    - CASE
- Support and isolation metrics, collected via survey at baseline and quarterly (to be evaluated as potential mediators of treatment effects)
    - PROMIS Informational Support 2.0
    - PROMIS Instrumental Support 2.0
    - Social isolation

Exploratory Objectives and Outcome Measures

Exploratory objective(s) serve as a basis for explaining or supporting findings of primary and secondary analyses and for suggesting further hypotheses for later research.

Exploratory effectiveness outcomes in NU IMPACT:

- To compare clinical endpoints for EC vs. UC

Exploratory effectiveness outcomes in NU IMPACT:

- Curative intent group
    - Recurrence
    - Progression-free survival
    - Overall survival

- Non-curative intent group
    - Secondary cancer(s)
    - Progression-free survival
    - Overall survival
- Survivorship group
    - Recurrence

All exploratory clinical outcomes within 1 year of eligibility will be ascertained via the electronic medical record.

3.0 STUDY DESIGN

Implementation Study

A schematic of the study design that illustrates the test of the implementation strategy package is depicted in Figure 1. All clusters will begin with a 6-month pre-I period of data collection. We will use a stepped-wedge trial design comprising seven total clusters. Clusters are the unit of randomization. We will use non-random assignment of the first cluster in the sequence, which was based on readiness to start on our proposed time table, followed by simple randomization of the remaining six clusters to the time of transition from pre-I to post-I. The seven clusters contain 32 clinics (1-9 clinics per cluster). The clinics comprising each of the clusters largely follow naturally occurring administrative structures within the health system (e.g., they share operational leadership). Possible times of transition from pre-I to post-I will follow a quarterly schedule. This will allow within- and between-site evaluation of system-level changes due to prospective introduction of implementation strategies in the observational group of patients completing cPRO assessments and being triaged and referred for relevant clinical services based on their assessment results, under UC. Importantly, the majority of implementation strategies within the package begin immediately prior to or at the point of each cluster's transition from pre-I to post-I.

	Year 3				Year 4				Year 5				Year 6			
	Q1	Q2	Q3	Q4	Q1	Q2	Q3	Q4	Q1	Q2	Q3	Q4	Q1	Q2	Q3	Q4
Cluster 1	pre-I	pre-I	post-I	post-I	post-I	post-I	post-I	post-I	post-I	post-I	post-I	post-I	post-I	post-I		
Cluster 2	pre-I	pre-I	pre-I	post-I	post-I	post-I	post-I	post-I	post-I	post-I	post-I	post-I	post-I	post-I		
Cluster 3	pre-I	pre-I	pre-I	pre-I	post-I	post-I	post-I	post-I	post-I	post-I	post-I	post-I	post-I	post-I		
Cluster 4	pre-I	pre-I	pre-I	pre-I	pre-I	post-I	post-I	post-I	post-I	post-I	post-I	post-I	post-I	post-I		
Cluster 5	pre-I	pre-I	pre-I	pre-I	pre-I	pre-I	post-I	post-I	post-I	post-I	post-I	post-I	post-I	post-I		
Cluster 6	pre-I	pre-I	pre-I	pre-I	pre-I	pre-I	pre-I	post-I	post-I	post-I	post-I	post-I	post-I	post-I		
Cluster 7	pre-I	pre-I	pre-I	pre-I	pre-I	pre-I	pre-I	pre-I	post-I	post-I	post-I	post-I	post-I	post-I		

Figure 1: Stepped Wedge trial design for evaluating implementation outcomes. After an initial 6 months with all 7 clusters contributing pre-I data, the first designated cluster transitions to post-I with the remaining 6 clusters randomly assigned to time points at quarterly intervals to transition from pre-I to post-I by introducing a system-level multicomponent package of implementation strategies for oncology symptom assessment and management pre-I: Light blue, post-I: Dark blue

Effectiveness Study

A schematic of the study design that illustrates the effectiveness investigation is depicted in Figure 2. For the last 6 months of each cluster's pre-I phase, eligible participants are invited to consent to monthly administration of a collection of PRO surveys administered through REDCap for one year following consent. One set of comprehensive PRO surveys will be administered at baseline and monthly thereafter, and an additional set of questionnaires will be included with these PRO surveys on a quarterly basis (see Section 2.0 for primary and secondary effectiveness PROs and timing). During post-I, EC becomes available using a 1:1 randomization scheme (EC:UC) to new, consenting patients, but not those in the consented group from the pre-I period who are continuing their care. Randomization will be stratified by cluster, cancer continuum (curative, non-curative, survivorship), gender and language preference. Participants who consent to randomization during post-I will receive the REDCap PRO surveys on the same schedule as those who consented during the pre-I phase. The monthly PRO data collected over one year of follow-up during the post-I embedded patient-randomized trial will be the primary data source for the effectiveness analysis. Additionally, we will compare the post-I EC and UC arms to patients enrolled during pre-I. Pre-I patients will have access to identical tools as UC patients, and the comparison of these arms will be critical for noting potential contamination in provider care subsequent to system-wide implementation strategies.

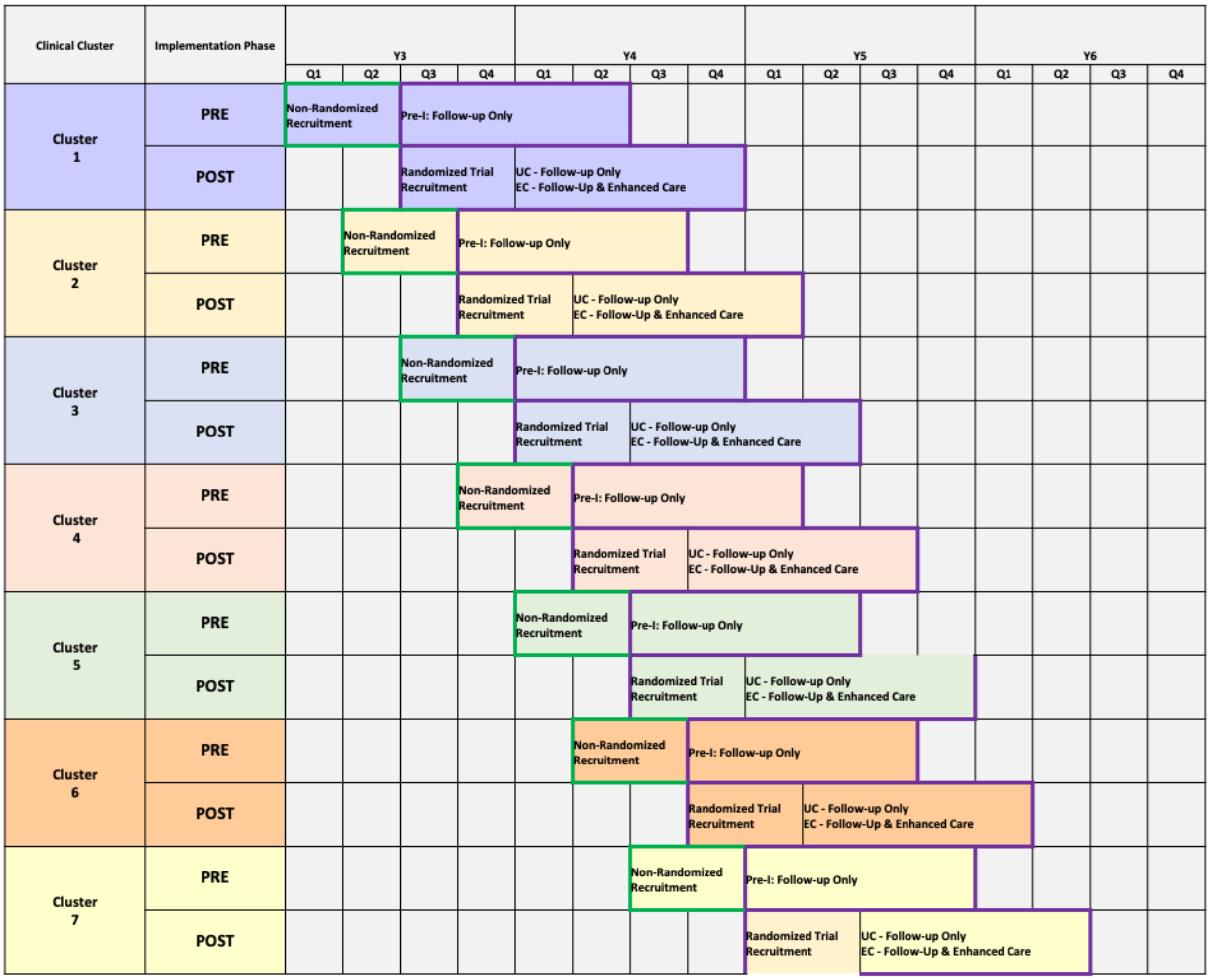

Figure 2: Patient-randomized trial design for evaluating effectiveness outcomes. During Pre-I, participants are recruited to consent to monthly PRO surveys administered through REDCap for one year following consent. During Post-I, participants are recruited to the same schedule of PRO surveys as in the pre-I phase and are additionally randomized 1:1 to EC or UC.

4.0 STUDY COHORT

Participant Inclusion Criteria – Implementation

To be eligible for the stepped wedge component of the NU IMPACT study to evaluate implementation outcomes, individuals must meet the following criteria:

- Cancer diagnosis in medical record (ICD-9/ICD-10 code on problem list or encounter diagnosis)
- Visit to the clinic during study period
- Still in care at NMHC (e.g., not deceased, care terminated, etc.)

Participant Inclusion Criteria – Effectiveness

To be eligible to participate in the NU IMPACT patient-randomized trial to evaluate effectiveness outcomes, an individual must meet all the following criteria. Recruitment to the patient-randomized study will take place through MyChart using data available in Northwestern Medicine Enterprise Data Warehouse (NMEDW), the integrated repository of clinical and research data for NMHC and NU FSM, to identify eligible patients.

- Cancer diagnosis in medical record (ICD-9/ICD-10 code on problem list or encounter diagnosis) within past 10 years
- Age >=18 years
- Has valid email address
- Provider agreed to patients being contacted
- Visit to clinic in the past year

Only data from participants who consent to monthly PRO data collection via REDCap during either pre-I or post-I will be used to evaluate effectiveness of EC.

5.0 STUDY HYPOTHESES

The *primary implementation hypothesis* is that the system-level multicomponent package of implementation strategies for oncology symptom assessment and management at NMHC will increase the cluster-level reach rate of cPRO to eligible patients. The *primary effectiveness hypothesis* is that cancer patients at NMHC who are randomized to EC will have better PRO outcomes over time compared to cancer patients at NMHC who are randomized to UC.

Secondary implementation hypotheses are that the system-level multicomponent package of implementation strategies for oncology symptom assessment and management at NMHC will increase adoption of cPRO at the provider level as evidenced by referrals for appropriate services when a patient triggers in cPRO and the reach rate of patients enrolling in the portal. It is also hypothesized that system-level strategies will be appropriate, acceptable, feasible, and sustainable. *Secondary effectiveness hypotheses* are that cancer patients at NMHC who are randomized to EC will have lower healthcare utilization, better cancer treatment delivery, better cancer treatment satisfaction, better health related quality of life, better self-management, lower toxicity, better physical health and lower financial burden compared to cancer patients at NMHC who are randomized to UC.

The *exploratory hypothesis* is that cancer patients randomized to EC will have better clinical outcomes compared to cancer patients randomized to UC.

6.0 SAMPLE SIZE CONSIDERATIONS

Implementation

It is anticipated that data gathered through the electronic health record will be available for approximately 12,000 patients to be used for the implementation analyses. This estimate is based on the sizes of the clusters of clinics that are involved in the NU IMPACT study. Based upon prior years' metrics, an annual volume of over 18,000 unique patients across the clinics is projected, ranging from 1,500-4,500 per cluster. We will study implementation of cPRO for all adult patients being treated in all three phases of the cancer treatment continuum. Although there is some cross-cluster variability in proportion of patients in each treatment phase, we estimate that on average each cluster will see 30% of its new patients being treated for cure, 30% will be treated for life extension, and 40% will be disease-free survivors. Based upon our prior experience with similar studies, we anticipate that approximately 2/3 of patients will meet the inclusion criteria listed above, hence the total anticipated 12,000.

Effectiveness

Target enrollment in the patient-randomized trial comparing EC v. UC in the post-I phase is approximately 1000 patients. The target enrollment for the patient-randomized component of the trial was based on planned longitudinal modeling of monthly evaluations of 5 PROs for anxiety, depression, fatigue, physical functioning and pain interference. Assuming a compound symmetry covariance structure for the longitudinal measurements with within-subject correlations of 0.5 at all time points, a sample size of 360 is required to achieve 90% power to achieve statistical significance at a Bonferroni-corrected $p<0.01$ for an anticipated average difference of 0.3 SD across all time points for EC v. UC. An overall recruitment goal of n=1000 was set to accommodate dropout of up to 64% of patients over time. A Bonferroni correction was used for a conservative estimate of required sample size; for statistical analyses the more powerful step-down Dunnett procedure will be used for p-value adjustment. It is also noted that the target sample size of n=1000 also provides greater than 90% power for detecting a 0.3 SD difference in the FACT G-7 (evaluated at 5 time points) under similar covariance assumptions as the other PROs, as well as simple change-from-baseline differences in PROs from 0 to 12 months using linear models.

7.0 DATA LOCK AND DESCRIPTIVE ANALYSES

7.1 Quality Assessment and Data Lock

Implementation

Data to be used for implementation analyses will be collected from the electronic health record using structured data queries. Variables will be created, summarized, and reviewed with study leadership to ensure appropriateness and clinical interpretability.

A final data set will be prepared for use in statistical analyses for implementation that will include observations that meet the following criteria:

- Confirmation of patient eligibility

Data from individuals who consent to monthly REDCap PRO surveys during Pre-I or randomization to EC or UC with monthly REDCap PRO surveys during Post-I will be removed from the implementation analysis data set.

Effectiveness

Throughout NU IMPACT conduct, REDCap data will be assessed biweekly for completeness. Balance of participant characteristics across treatment arms will also be assessed.

At the completion of NU IMPACT recruitment and follow-up, and after final cleaning and query resolution, data will be locked.

A final data set will be prepared for use in statistical analyses for effectiveness that will include observations that meet the following criteria:

- Confirmation of eligibility, consent and study enrollment
- Completion of at least one baseline PRO measure and at least one additional PRO measure over the course of follow-up

7.2 Descriptive Analyses

Implementation

Prior to formal statistical analyses that evaluate implementation outcomes, we will compare patient characteristics (cancer type, cancer treatment continuum, race/ethnicity, age, and gender) on each outcome of interest (e.g., cPRO enrollment, cPRO completion) stratified by pre-I and post-I, for the full sample, and within each cluster.

Effectiveness

Prior to formal statistical analyses that evaluate effectiveness outcomes, we will first summarize demographic and baseline characteristics across study treatment arms to gauge comparability of Pre-I participants who consented to REDCap PRO surveys, as well as patients randomized to UC or EC during post-I. For each cancer treatment stratum (curative, non-curative, survivorship), we will calculate summary statistics for variables including gender, language preference, age, race/ethnicity, cancer diagnosis, and cancer stage. Means and standard deviations or medians and interquartile ranges will be used for continuous variables. Tables of frequencies and counts will be used for categorical variables. Summaries will be calculated both within each cluster and collapsed across all clusters. We expect baseline characteristics to be comparable across Pre-I, UC and EC groups since all eligible patients will be approached for participation in the study in both the pre- and post-implementation phases, and since patients will be randomized 1:1 to UC or EC. It is expected that stratified randomization in the patient-randomized trial will ensure balance of the treatment arms across cluster, cancer continuum, gender, and language preference.

For final reporting of effectiveness results, the following clinical and demographic variables will be included in descriptive summaries: NMHC cluster, cancer care continuum strata (curative, non-curative, survivorship), preferred language, participant's gender, age at enrollment and self-reported race/ethnicity. Primary and secondary outcomes will also be tabulated and summarized for manuscripts that are prepared to address these outcomes. Other clinical and demographic variables may also be included for description of the NU IMPACT study population.

8.0 STATISTICAL ANALYSES - IMPLEMENTATION

8.1 Primary Outcome - Implementation

Reach (proportion of patients)

Reach will be measured by the proportion of patients completing one or more (≥1) cPRO assessments among those eligible for cPRO. This analysis will be a clinic-level analysis analyzed under a cross-sectional design and will only include clinics in the 6 clusters that were randomized. We will calculate reach using 1-month sampling periods within each clinic (as less than 100 individuals in a clinic would be too small for a per-month analysis, our back up will be a 2-month sampling period rather than a 1-month sampling period). The outcome variable will be defined as the proportion of participants who complete a cPRO assessment among those who are eligible within the clinic and the 1-month period. Thus, each month and clinic will have a single data point expressed as a proportion of eligible individuals reached. There are approximately 30 clinics (median of 5 clinics per cluster) and 42 monthly reach proportions will be calculated for each clinic; thus we anticipate 1260 data points for the primary outcome variable. We will use the nlme package in R to perform generalized least squares linear regression modeling with an exposure indicator variable for post-I v. pre-I [referent] entry into the study as the primary predictor of interest. An autoregressive structure will be specified for the correlation structure among the linear model residuals resulting from the repeated measurements of reach within clinic; a lag 1 model will be specified, but model fit under this and other correlation structure specifications will be evaluated according to AIC and BIC. In the generalized least squares setting, the within-clinic repeated measures observation structure is used to define the structure of the covariance matrix. The degrees of freedom is thus the number of calculated proportions calculated for all 1-month intervals and all clinics, less the number of model parameters, including those specified in the covariance matrix. We will perform analyses using an identity link for the outcome variable to estimate risk differences on the raw proportions. In addition, we will log-transform the outcomes to estimate relative risk ratios. We will use clinic-level covariate adjustment for: 1) cluster; 2) time (categorical variable for quarter); 3) number of 1.0 FTE equivalent oncologists per clinic; 4) number of unique patients per clinic; and 5) type of cancer care clinic (e.g., medical oncology, surgical oncology). In sensitivity analyses, we will use weights in the generalized least squares model based on the number of unique eligible patients per clinic. Restricted maximum likelihood (REML) will be used for model parameter estimation as it produces unbiased estimates of the variance and covariance parameters. The primary predictor of interest will be post-I v. pre-I entry into the study, i.e. whether the individual became eligible for cPRO during the pre-I or post-I phase at their site. This clinic-level analysis was chosen as we hypothesize that the impact of the implementation strategy will vary by clinic due to clinic size (clinicians and patients). We will also test for cluster*treatment and time*treatment effects to test for a lagged/delayed effect of the exposure, and clinic effects within clusters, in the overall model. Rather than including an a priori transition period, we will conduct a sensitivity analysis around the crossover point in two 1-month increments.

8.2 Secondary Outcomes - Implementation

Reach (proportion of patients)
Secondary reach outcomes will be measured as the proportion of patients enrolled in the patient cPRO portal among those eligible and the proportion of patients who are referred for appropriate services from among those that trigger an alert in cPRO. Similar to the primary reach outcome, these proportions will be defined at the clinic level for each 1-month period. The analytic framework for these clinic-level outcomes will be the same as what is described for the primary reach outcome (Section 8.1).

Adoption (provider-level)
Adoption by providers will be measured by the proportion of clinicians within a clinic who refer ≥1 patient who trigger an alert for services, using a cPRO dot phrase, within each 1-month period. The analytic framework for these clinic-level outcomes will be the same as what is described for the primary reach outcome, but with the proportion outcome defined at the provider level (Section 8.1). We will perform a cluster-level analysis if there are fewer than 20 clinicians in any one clinic.

Survey Outcomes for Appropriateness, Acceptability, Feasibility, and Sustainability

Survey summary scores will be computed according to established methods in the literature concerning full/subscale scores, the metric reported, etc. We will examine score differences between respondents by provider/respondent type, associated cluster, and the other clinician covariates previously specified.

9.0 STATISTICAL ANALYSES - EFFECTIVENESS

9.1 Primary Outcomes - Effectiveness

PROs

Using each of the separate PROMIS domain status scores (i.e., Anxiety, Depression, Fatigue, Pain Interference, and Physical Function) collected at baseline and at regular monthly intervals, primary analyses will use longitudinal models to estimate the time-averaged difference in PROs across groups. Primary effectiveness estimates will be based on post-I data with a comparison of longitudinal PROs for patients randomized to Post-I EC v. Post-I UC. Linear mixed models will be used for these analyses with a fixed effect for treatment arm (Post-I EC v. Post-I UC) as the primary predictor of interest and fixed effects for model adjustments for cluster, cancer continuum, gender, language preference, baseline PRO value and time of enrollment (categorical variable for quarter enrolled). Individual-level random effects with an autoregressive covariance structure will be included in the mixed models to account for dependence of within-individual PRO measurements over time. Multiple comparisons adjustment for the five primary tests of hypothesis will be performed using the step-down Dunnett procedure[5]. For reporting purposes, confidence intervals for treatment effects for the five primary outcomes will be reported with lower and upper bounds simultaneously estimated to correspond with Dunnett correction[6,7]. Sensitivity of model estimates and statistical inference under unstructured and compound symmetry covariance structures will be explored. In addition to the overall time-averaged difference, we will also report estimated differences from baseline at specific monthly timepoints.

Secondary analyses will also examine changes from baseline to the 12-month follow-up time point as well as change from baseline to the other monthly follow-up time points using the directly observed differences from baseline at these timepoints. Linear regression will be used for these analyses with treatment arm (Post-I EC v. Post-I UC) as the primary predictor of interest and model adjustments for cluster, cancer continuum, gender, language preference, baseline PRO value and time of enrollment (categorical variable for quarter enrolled). These effect estimates will be compared to effect estimates at each time point calculated in the primary analysis model that uses all longitudinal measurements.

Recognizing the possibility of contamination effects for the treatment arms during the post-I period, we will augment the primary analyses to include longitudinal PRO data from the Pre-I period. Linear mixed models will be used as described for the primary analyses but extending the primary predictor of interest to a multicategory variable (Post-I EC, Post-I UC, Pre-I). Adjustments for cluster, cancer continuum, gender, language preference, baseline PRO value and time of enrollment, and the individual-level random effects, will be included as described above. The Post-I UC cohort will be treated as the referent group to facilitate formal primary comparison with outcomes for the Post-I EC group, and to gauge comparability of outcomes for Post-I UC and Pre-I patients. This approach will also allow for a contrast test between Post-I EC and Pre-I. If longitudinal PROs for Post-I UC and Pre-I are not significantly different, and if the effectiveness estimate for Post-I EC v. Pre-I is similar to the primary Post-I EC v. Post-I UC comparison, this will suggest lack of contamination. If longitudinal PRO differences for Post-I UC v. Pre-I are significantly different and/or the effectiveness estimate for Post-I EC v. Pre-I is different from the primary Post-I EC v. Post-I UC comparison, this may suggest some level of treatment arm contamination during the Post-I phase. This latter scenario may also be indicative of system-level implementation effects and will need to be interpreted in the context of implementation analysis findings.

Multiple imputation using chained equations will be used for missing data under a range of scenarios from missing at random to informative missingness [8]. Sensitivity of parameter estimates to these assumptions will be evaluated.

9.2 Secondary Outcomes – Effectiveness

Healthcare Utilization
Healthcare utilization metrics include the numbers of ER/urgent care visits, hospital admissions and days of stay, unscheduled visits, and supportive care visits for all three curative, non-curative and survivorship groups across the follow-up time frame for each individual. These data will be captured through the electronic medical record for within-NMHC utilization and by data collection directly from patients for outside-NMHC utilization at baseline, 6 and 12 months. Individual variables will be summarized across treatment groups using means and five-number summaries (minimum, 25th percentile, median, 75th percentile, maximum). For formal statistical analysis, we intend to create a global summary score of healthcare utilization by summing the counts of all variables listed above. Poisson regression will be used to compare the global healthcare utilization summary measures at 6- and 12-month time points across treatment arms in separate analyses for each time point. Primary analyses will compare Post-I EC v. Post-I UC as the primary predictor of interest with model adjustments for cluster, cancer continuum, gender, language preference, and time of enrollment. To evaluate potential contamination effects as described above, differences across the Post-I EC, Post-I UC and Pre-I groups will also be jointly evaluated. Overdispersion will also be evaluated, and if present, will be addressed through the use of quasi-Poisson modeling to account for additional variance.

Cancer Treatment Delivery
Cancer treatment delivery metrics include treatment satisfaction, number of treatment breaks, and number of changes in regimen. Cumulative numbers of treatment breaks and changes in regimen over the follow-up time frame will be summarized across treatment arms and compared using the Poisson regression approach described above for healthcare utilization outcomes.

Cancer Treatment Delivery Satisfaction, Health Related Quality of Life, Self-Management, Symptoms and Toxicities, Physical Activity, Financial Burden
All of these additional secondary effectiveness outcomes will be measured using the PRO measures described above over regular intervals for each cohort. The analytic methodology described above for PRO data analysis will be applied here.

Support and Isolation
Social support and isolation metrics will be evaluated as potential mediators of treatment effects on primary outcome PROs. Structural equation modeling of potential mediation effects on the longitudinal PRO outcomes will be implemented using functionality in the lavaan R package.

9.3 Exploratory Objectives

Heterogeneity of cancer diagnoses for patients in the NU IMPACT trial makes formal analysis of clinical outcomes a challenge. However, we will undertake exploratory analyses of clinical outcomes to assess differences in the Pre-I, Post-I UC and Post-I EC arms. Clinical endpoints to be ascertained via the electronic medical record through 12 months of follow-up include the following for each cancer continuum cluster: curative intent – recurrence, secondary cancers, disease-free and overall survival; non-curative intent – secondary cancers, progression-free and overall survival; survivorship – recurrence. Kaplan-Meier curves will be used to estimate distributions for time-to-event outcomes disease-free, progression-free and overall survival within treatment arm, and global differences across arms will be compared using logrank tests. Cox proportional hazards regression models will be used to estimate hazard ratios, treating the Pre-I cohort as the referent. Frequencies of binary outcomes recurrence and secondary cancers will be summarized across treatment arms

and compared using chi-square tests. Logistic regression models will be used to estimate odds ratios, treating the UC cohort as the referent with global and pairwise comparisons for Post-I EC v. Post-I UC and Pre-I v. Post-I UC. Regression models will be adjusted for cluster, cancer continuum, gender, language preference, and time of enrollment. If differences in demographic or baseline variables are detected across treatment arms, we will evaluate sensitivity of estimated hazard and odds ratios to further adjustment for these variables. Importantly, given the heterogeneity of outcome rates for different cancers, we will conduct these analyses separately for each type of cancer diagnosis. Given the subset nature of these investigations, these analyses will be considered exploratory.

10.0 SOFTWARE

All statistical analyses will be conducted using R statistical software in a reproducible R Markdown format. Specific R packages and versions will be documented and reported.

REFERENCES

1. Cella D, Choi S, Garcia S, et al. Setting standards for severity of common symptoms in oncology using the PROMIS item banks and expert judgment. *Qual Life Res*. 2014;23(10):2651-61. doi:http://dx.doi.org/10.1007/s11136-014-0732-6
2. Wagner LI, Schink J, Bass M, et al. Bringing PROMIS to practice: Brief and precise symptom screening in ambulatory cancer care. *Cancer*. 2015;121(6):927-34. doi:https://www.ncbi.nlm.nih.gov/pmc/articles/PMC4352124/
3. Yost KJ, Eton DT, Garcia SF, Cella D. Minimally important differences were estimated for six Patient-Reported Outcomes Measurement Information System-Cancer scales in advanced-stage cancer patients. *J Clin Epidemiol*. 2011;64(5):507-16. doi:http://dx.doi.org/10.1016/j.jclinepi.2010.11.018
4. Wagner L, King K, Kozlowski C, et al. Integrating a Web-based Assessment to Identify Distress Into Routine Cancer Care: Clinical Application of the Nih Patient Reported Outcomes Measurement Information System Computer Adaptive Test. *Psychooncology*. 2013;22:7.
5. Naik UD. Some selection rules for comparing p processes with a standard. *Communications in Statistics, Series A*. 1975;4:519-535.
6. Bofinger E. Step-down procedures for comparison with a control. *Australian Journal of Statistics*. 1987;29:348-364.
7. Stefansson G, Kim W-C, Hsu JC. On confidence sets in multiple comparisons. In: Gupta SS, Berger JO, eds. *Statistical Decision Theory and Related Topics IV*. Academic Press; 1988:89-104.
8. Van Buuren S, Groothuis-Oudshoorn K. mice: Multivariate Imputation by Chained Equations in R. *Journal of Statistical Software*. 2011;45(3):1-67.
