# Supplementary material for: Web-Based Cancer Symptom Self-Management System: A Randomized Clinical Trial
Source: JAMA Netw Open. 2025 May 5;8(5):e258353. doi: 10.1001/jamanetworkopen.2025.8353 (PMC12053558; doi:10.1001/jamanetworkopen.2025.8353)
Supplement: Supplement 2. — eTable 1. Mean Changes in PROMIS Scores From Baseline to 6 and 12 Months eTable 2. Primary Outcome Results from Linear-Mixed Model: Including Preimplementation eTable 3. Mean Numbers Completed Over Study Period by Group eTable 4. Primary Outcome Numbers Completed at Each Time Point by Group eTable 5. Health Care Utilization Outcomes and Treatment Effects at 6 and 12 Months eTable 6. Primary Outcome Results From Linear-Mixed Models Stratified by Beacon Intent eFigure. Proportion Completed at Each Time Point by Group [file jamanetwopen-e258353-s002.pdf]

Supplementary Online Content

Cella D, Lancki N, Kuharic M, et al. Web-based cancer symptom self-management system: a randomized clinical trial. *JAMA Netw Open.* 2025;8(5):e258353. doi:10.1001/jamanetworkopen.2025.8353

eTable 1. Mean Changes in PROMIS Scores From Baseline to 6 and 12 Months

eTable 2. Primary Outcome Results from Linear-Mixed Model: Including Preimplementation

eTable 3. Mean Numbers Completed Over Study Period by Group

eTable 4. Primary Outcome Numbers Completed at Each Time Point by Group

eTable 5. Health Care Utilization Outcomes and Treatment Effects at 6 and 12 Months

eTable 6. Primary Outcome Results From Linear-Mixed Models Stratified by Beacon Intent

eFigure. Proportion Completed at Each Time Point by Group

This supplementary material has been provided by the authors to give readers additional information about their work.

© 2025 Cella D et al. *JAMA Network Open*.

eTable 1. Mean Changes in PROMIS Scores From Baseline to 6 and 12 Months

PRO Domain	Enhanced Care		Usual Care		Estimate (95% CI) for Treatment Effect [b] (EC vs UC)	P value
	**Baseline** Mean (SD)[a]	**6 Months** Mean (SD)[a]	**Baseline** Mean (SD)[a]	**6 Months** Mean (SD)[a]		
Depression T Score	49.4 (8.3)	46.9 (8.7)	49.3 (8.3)	47.0 (8.9)	-0.11 (-0.85, 0.62)	0.76
Anxiety T Score	51.8 (9.2)	48.7 (9.3)	52.0 (9.4)	49.1 (9.8)	0.17 (-0.61, 0.96)	0.67
Physical Function T Score	46.5 (9.0)	46.2 (8.9)	46.4 (8.6)	46.5 (8.5)	0.31 (-0.34, 0.97)	0.35
Fatigue T Score	50.7 (10.2)	50.1 (10.2)	50.8 (10.3)	49.6 (10.4)	-0.26 (-1.17, 0.64)	0.57
Pain Interference T Score	50.2 (9.6)	49.7 (9.9)	50.2 (9.6)	49.0 (9.9)	-0.40 (-1.34, 0.55)	0.41

PRO Domain	Enhanced Care		Usual Care		Estimate (95% CI) for Treatment Effect [b] (EC vs UC)	P value
	**Baseline** Mean (SD)[a]	**12 Months** Mean (SD)[a]	**Baseline** Mean (SD)[a]	**12 Months** Mean (SD)[a]		
Depression T Score	49.4 (8.3)	46.9 (8.9)	49.3 (8.3)	46.7 (8.9)	-0.09 (-0.84, 0.65)	0.80
Anxiety T Score	51.8 (9.2)	48.3 (9.2)	52.0 (9.4)	48.7 (9.8)	0.09 (-0.69, 0.86)	0.82
Physical Function T Score	46.5 (9.0)	46.4 (8.8)	46.4 (8.6)	46.4 (8.8)	0.54 (-0.14, 1.23))	0.20
Fatigue T Score	50.7 (10.2)	49.1 (10.6)	50.8 (10.3)	49.4 (10.7)	0.05 (-0.86, 0.97)	0.91
Pain Interference T Score	50.2 (9.6)	49.5 (9.9)	50.2 (9.6)	49.1 (9.8)	-0.25 (-1.19, 0.69)	0.60

Abbreviations: PRO=patient reported outcome, CI=Confidence Interval, EC=Enhanced Care, UC=Usual Care

[a] Mean (SD) are from observed values

[b] From model adjusted for gender, Beacon therapeutic intent category, cluster, study language, baseline PRO value and quarter enrolled

© 2025 Cella D et al. *JAMA Network Open*.

eTable 2. Primary Outcome Results from Linear-Mixed Model: Including Preimplementation

	**Post-I EC vs Post-I UC**		**Pre-I UC vs Post-I UC**	
**PRO Domain**	**Estimate [a] (SE)**	**P value**	**Estimate [a] (SE)**	**P value**
Depression T Score	-0.09 (0.20)	0.64	0.49 (0.32)	0.13
Anxiety T Score	0.14 (0.21)	0.50	0.19 (0.35)	0.59
Physical Function T Score	0.20 (0.18)	0.27	0.02 (0.29)	0.93
Fatigue T Score	-0.09 (0.29)	0.74	-0.16 (0.44)	0.72
Pain Interference T Score	-0.02 (0.24)	0.93	0.23 (0.40)	0.57

Abbreviations: Post-I=Post-Implementation, Pre-I=Pre-Implementation, EC=Enhanced Care, UC=Usual Care, PRO=patient reported outcome, SE=Standard Error

[a] From model adjusted for gender, Beacon therapeutic intent category, cluster, study language, baseline PRO value and quarter enrolled

eTable 3. Mean Number of Assessments Completed Over Study Period by Group

**Characteristic**	**Enhanced Care (n = 731)**	**Usual Care (n = 716)**
Number of Timepoints Completed Any		
Mean (SD)	9.7 (3.9)	9.7 (3.8)
Median (IQR)	12 (7, 13)	11 (7, 13)
[Range]	[1, 13]	[1, 13]
Number of Timepoints Completed All		
Mean (SD)	9.6 (3.9)	9.5 (3.9)
Median (IQR)	11 (6, 13)	11 (7, 13)
[Range]	[1, 13]	[1, 13]

Abbreviations: SD=standard deviation, IQR=Interquartile Range

© 2025 Cella D et al. *JAMA Network Open*.

eTable 4. Primary Outcome Numbers Completed at Each Time Point by Group

Study Month	Number Completed Any domain: EC	Number Completed Any domain: UC	Number Completed All 5 domains [a]: EC	Number Completed All 5 domains [a]: UC
**0**	699	662	692	654
**1**	639	619	631	610
**2**	620	602	610	596
**3**	595	581	585	573
**4**	564	557	552	550
**5**	546	540	539	530
**6**	539	535	533	523
**7**	508	504	501	491
**8**	491	498	480	489
**9**	481	470	477	466
**10**	469	453	465	447
**11**	463	449	459	441
**12**	465	465	462	458

Abbreviations: EC=Enhanced Care, UC=Usual Care

[a] The 5 Patient Reported Outcome Domains collected: Depression, Anxiety, Physical Functioning, Fatigue, Pain Interference

© 2025 Cella D et al. *JAMA Network Open*.

eTable 5. Health Care Utilization Outcomes and Treatment Effects at 6 and 12 Months

**6 Months**						
**Outcome**	**Enhanced Care**		**Usual Care**		**IRR (95% CI) for Treatment Effect [a] (EC vs UC)**	**P value**
	**n**	**# outcome/Person-Years observed (Rate)**	**n**	**# outcome/Person-Years observed (Rate)**		
Global Summary [b]	792	816/393.2 (2.08)	800	801/395.7 (2.02)	1.05 (0.89, 1.26)	0.55
Hospital Admissions	792	580/393.2 (1.48)	800	556/395.7 (1.41)	0.94 (0.72, 1.22)	0.64
ER/UC visits	792	236/393.2 (0.60)	800	245/395.7 (0.62)	1.10 (0.92, 1.33)	0.30
Hospital days	792	1510/393.2 (3.84)	800	1315/395.7 (3.32)	1.02 (0.77, 1.34)	0.91
Any OTC clinic visits [c]	316	5/157.1 (0.03)	320	2/157.9 (0.01)	2.21 (0.44, 16.2)	0.36

**12 Months**						
**Outcome**	**Enhanced Care**		**Usual Care**		**IRR (95% CI) for Treatment Effect [a] (EC vs UC)**	**P value**
	**n**	**# outcome/Person-Years observed (Rate)**	**n**	**# outcome/Person-Years observed (Rate)**		
Global Summary [b]	792	1344/774.4 (1.74)	800	1417/780.7 (1.82)	0.96 (0.83, 1.12)	0.63
Hospital Admissions	792	957/774.4 (1.24)	800	1007/780.7 (1.29)	0.90 (0.72, 1.12)	0.35
ED/UC visits	792	387/774.4 (0.50)	800	410/780.7 (0.53)	0.99 (0.84, 1.16)	0.88
Hospital days of stay	792	2279/774.4 (2.94)	800	2319/780.7 (2.97)	1.05 (0.83, 1.33)	0.65
Any OTC clinic visits [c]	316	5/307.0 (0.02)	320	2/309.1 (0.01)	2.21 (0.44, 16.20)	0.36

Abbreviations: IRR=Incidence Rate Ratio, CI=Confidence Interval, EC=Enhanced Care, UC=Usual Care, ED = emergency department, UC (outcome) = Urgent Care, OTC = Oncology Triage Clinic

[a] From model adjusted for gender, Beacon therapeutic intent category, cluster, study language and quarter enrolled

[b] Global summary is the sum of Hospital Admissions and ED/Urgent Care visits

[c] Estimate for this row is odds ratio (95% CI). The 6-month and 12-month estimates are identical as all OTC visits occurred within the first 6 months of the study period

© 2025 Cella D et al. *JAMA Network Open*.

eTable 6. Primary Outcome Results From Linear-Mixed Models Stratified by Beacon Intent

	Beacon Therapeutic Intent					
	Curative		Non-Curative		No Beacon Plan	
**PRO Domain**	**Estimate [a] (95% CI) for treatment effect (EC vs UC)**	**P value**	**Estimate [a] (95% CI) for treatment effect (EC vs UC)**	**P value**	**Estimate [a] (95% CI) for treatment effect (EC vs UC)**	**P value**
Depression T Score	0.85 (0.02, 1.68)	0.05	-0.68 (-1.89, 0.52)	0.26	0.02 (-0.45, 0.49)	0.93
Anxiety T Score	0.69 (-0.22, 1.59)	0.14	-0.50 (-1.78, 0.79)	0.45	-0.29 (-0.79, 0.22)	0.27
Physical Function T Score	-0.11 (-0.89, 0.66)	0.77	0.05 (-0.90, 1.00)	0.92	-0.22 (-0.68, 0.24)	0.35
Fatigue T Score	0.91 (-0.17, 1.99)	0.10	-0.03 (-1.39, 1.32)	0.96	-0.07 (-0.66, 0.51)	0.81
Pain Interference T Score	0.89 (-0.26, 2.04)	0.13	0.15 (-1.27, 1.58)	0.83	-0.26 (-0.84, 0.33)	0.39

Abbreviations: PRO=Patient Reported Outcome, CI=Confidence Interval, EC=Enhanced Care, UC=Usual Care
[a] Estimate is the time-averaged difference (baseline to 12 months) in T score for EC vs UC from model adjusted for gender, Beacon therapeutic intent category, cluster, study language, baseline PRO value and quarter enrolled

© 2025 Cella D et al. *JAMA Network Open*.

eFigure. Proportion of Completed Assessments at Each Time Point by Group

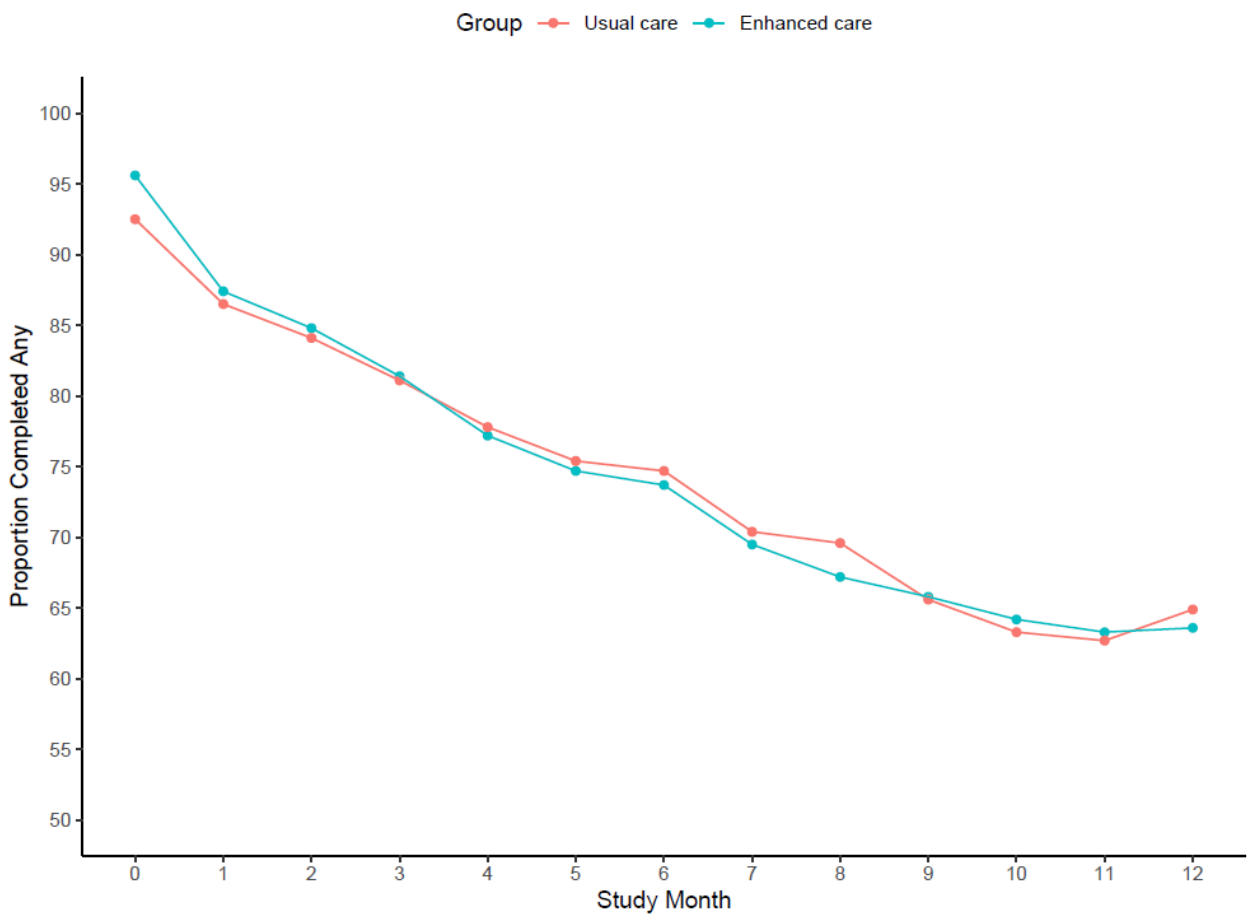

Proportion of participants who completed one or more PRO domain at each month stratified by group randomized to in the study. PROs were administered monthly via email as REDCap surveys. The denominator is among those included in the primary effectiveness analysis (n=731 for Enhanced Care, n=716 for Usual Care).

© 2025 Cella D et al. *JAMA Network Open*.
