# Supplementary material for: Web-Based Cancer Symptom Self-Management System: A Randomized Clinical Trial
Source: JAMA Netw Open. 2025 May 5;8(5):e258353. doi: 10.1001/jamanetworkopen.2025.8353 (PMC12053558; doi:10.1001/jamanetworkopen.2025.8353)
Supplement: Supplement 3. — Data Sharing Statement [file jamanetwopen-e258353-s003.pdf]

Data Sharing Statement

Cella. Web-Based Cancer Symptom Self-Management System. *JAMA Netw Open*. Published May 05, 2025. doi:10.1001/jamanetworkopen.2025.8353

Data
Additional Information: Trial Registration: ClinicalTrials.gov Identifier: NCT03988543 Trial protocol: The trial protocol has been published and is available at https://doi.org/10.1016/j.cct.2023.107171
Data available: Yes
Data types: Deidentified participant data, Data dictionary
How to access data: https://dataverse.harvard.edu/dataverse/HealthMeasures
When available: With publication

Supporting Documents
Document types: None

Additional Information
Who can access the data: with publication
Types of analyses: for any purpose
Mechanisms of data availability: with investigator support
